# Titanium and Protein Adsorption: An Overview of Mechanisms and Effects of Surface Features

**DOI:** 10.3390/ma14071590

**Published:** 2021-03-24

**Authors:** Jacopo Barberi, Silvia Spriano

**Affiliations:** Department of Applied Science and Technology, Politecnico di Torino, 10129 Turin, Italy; silvia.spriano@polito.it

**Keywords:** titanium, protein adsorption, biomaterials, surface modifications, cell interactions

## Abstract

Titanium and its alloys, specially Ti6Al4V, are among the most employed materials in orthopedic and dental implants. Cells response and osseointegration of implant devices are strongly dependent on the body–biomaterial interface zone. This interface is mainly defined by proteins: They adsorb immediately after implantation from blood and biological fluids, forming a layer on implant surfaces. Therefore, it is of utmost importance to understand which features of biomaterials surfaces influence formation of the protein layer and how to guide it. In this paper, relevant literature of the last 15 years about protein adsorption on titanium-based materials is reviewed. How the surface characteristics affect protein adsorption is investigated, aiming to provide an as comprehensive a picture as possible of adsorption mechanisms and type of chemical bonding with the surface, as well as of the characterization techniques effectively applied to model and real implant surfaces. Surface free energy, charge, microroughness, and hydroxylation degree have been found to be the main surface parameters to affect the amount of adsorbed proteins. On the other hand, the conformation of adsorbed proteins is mainly dictated by the protein structure, surface topography at the nano-scale, and exposed functional groups. Protein adsorption on titanium surfaces still needs further clarification, in particular concerning adsorption from complex protein solutions. In addition, characterization techniques to investigate and compare the different aspects of protein adsorption on different surfaces (in terms of roughness and chemistry) shall be developed.

## 1. Introduction

Almost 1000 tons of titanium-based biomaterials are worldwide used every year as orthopedic and dental implants [1] mainly as commercially pure titanium (cp-Ti) and αβ-alloy Ti6Al4V (Ti64), eventually as extra low interstitial (ELI) [2]. Despite titanium’s exceptional biocompatibility implant failure is a current issue. Dental and orthopedic implants have a failure rate ranging from 5 to 10% after up to 15 years [3]. Implant osseointegration is mediated by a complex series of events included in the immune response that are triggered as soon as the tissue are damaged during the surgery. The implant surface is immediately covered by a layer of water molecules and on top of them, a layer of protein is adsorbed within the first few minutes. That, along with cytokines released from the damaged cells and protein-promoted blood coagulation, triggers the foreign body reaction (FBR) of a host body to an implant [4,5]. In order to achieve osseointegration of Ti implants, an equilibrium within the events that occurs during FBR need to be found, in order to avoid chronic inflammation and fibrotic encapsulation. Concerning the inflammatory response, neutrophilis and monocytes are recruited at the implant site. Then (after about 48 h) monocytes differentiate into macrophages with a phagocytic function for controlling the immune response. In order to remove larger debris, monocytes and macrophages can fuse together forming foreign body giant cells (FBGCs). Concerning osseointegration, it takes place when mesenchymal stem cells (MSC) differentiate into osteoblasts and osteocytes, leading to new bone formation and it is triggered by an early pro-inflammatory response. When this does not happen, the worst scenario is proliferation of fibroblasts and the formation of a fibrous capsule that engulfs the implant, preventing a correct contact with the bone tissue [4,5]. Formation of the blood clot on the implant surface is of great importance to get the mechanical and biochemical environment for osseointegration. Activation of platelets within the blood clot on the implant surface provides a natural gradient of signal molecules with a high concentration on the surface of the implant and consequent attraction of monocytes, neutrophilis, and mesenchymal cells. This allows the “contact osteogenesis” mechanism with formation of immature bone characterized by irregularly arranged, interwoven collagenous fibers, and, as last, of mature lamellar bone through bone remodeling. Due to this cascade of events, cells do not interact directly with the implant surface, but the interface is strongly mediated and controlled by the adsorbed proteins. Cells exhibit specific binding sites for certain proteins and their activity can be enhanced by those adsorbed onto the material surface. For example, protein such as fibronectin (FN) and vitronectin (VN) are considered adhesive proteins. FN can activate cell α_5_β_1_ integrins through the binding domain arginine-glycine-aspartic acid (RGD), which is present in VN as well [6]. Bone morphogenetic (BMP) proteins can promote osseointegration [7] but contemporary increase the inflammatory response to biomaterials [8]. As a consequence, the ability to control the formation of the protein layer, by promoting selective adsorption, is of great interest in the design of novel and more functional biomaterials. Since the ‘70s of the past century, a great amount of efforts have been put in studying protein adsorption [9]. What happens when a surface gets in contact with a protein containing solution is dictated by a multitude of different factors. According to surface characteristics (roughness, wettability, charge), protein properties (surface charge, hydrophilicity, structure) [9], and even solution parameters (composition, pH, temperature) [10], adsorption of proteins may be driven by hydrophobic interactions, electrostatic attraction, or weak forces, such as Van deer Waals’ [11]. Furthermore, the protein layer is a dynamic entity, where adsorbed proteins can be displaced and replaced by the ones still in solution, and according to the so-called Vroman effect, they can change their conformation on the surface, spreading and reorienting themselves, and multilayers of loosely bound proteins can form thanks to protein–protein interactions [12]. Many authors have gathered and reviewed knowledge about driving forces of protein adsorption and their behavior on the surface [12,13,14]. Nevertheless, despite all these efforts, a fully and comprehensive understanding of adsorption processes and mechanisms is still missing. Even though we found that the protein adsorption on certain biomaterials was reviewed, such as bioactive glasses [15], bioceramics [16], metals of medical interest, steel [17], and magnesium [18], or even nanomaterials for biosensors [19], to our best knowledge, no work of this kind was made for protein adsorption on titanium. This review aims to put together the literature about adsorption of protein on titanium-based materials for osseointegration, trying to depict a comprehensive picture of how surface characteristics affect the way proteins bond to Ti surfaces. After a brief introduction on the main driving forces of protein adsorption, we focused on the principal characteristics of titanium surfaces. The literature was sorted accordingly to how titanium surfaces were treated or modified (kind of surface modification or activation, surface chemical composition, and crystalline structure) in order to explore the specific effect of the different surface features (wettability, roughness, hydroxylation, or charge) on protein adsorption. The same rational was applied to investigate how external parameters can affect the protein–titanium interaction and the correlations among surface features and the competitive adsorption of proteins. This knowledge can lead to an improved design of novel and more effective biomaterials. At last, the characterization techniques applied for studying protein adsorption on titanium-based biomaterials are collected. Due to the great amount of work published on these materials, only adsorption on bulk materials, these being pure Ti, Ti alloys, or titanium oxides (native or modified), is discussed. Adsorption on titania nanoparticles, as well as interaction of proteins with coated or functionalized surfaces were not included unless of interest for the selected topic. Relevant literature of the past 15 years was researched and selected.

## 2. Driving Forces and Factors Affecting Protein Adsorption

### 2.1. Driving Forces of Protein Adsorption

Protein adsorption is a complex phenomenon that involves different kinds of protein-substrate interactions and it is influenced by numerous different factors depending on the surfaces features and chemical or biological environment. 

It is well acknowledged that protein adsorption can be promoted by several kind of driving forces, these being hydrophobic or electrostatic interactions and van der Waals forces [12,20]. 

Hydrophobic interactions are fundamental in order to maintain the protein tertiary structure: When a protein is dissolved into a polar solvent, non-polar amino acid residues have the tendency of interact between each other, in the inside of the protein, to limit interaction with solvent. On the reverse, hydrophilic residues are exposed on the protein surface [21]. If a protein approaches a hydrophobic surface, the balance of protein–solvent and intra-protein interactions is disrupted, and hydrophobic residues can interact with the hydrophobic surface. Changes in protein structure lead to entropy gain, with this being a strong driving force for protein adsorption [22]. As a general rule of thumb, proteins adsorbed onto the hydrophobic surfaces undergo greater denaturation than on the hydrophilic ones [10]. Furthermore, dehydration of a surface has to happen for proteins to adsorb. On hydrophobic substrates, this results in reduction of Gibb’s free energy and increases protein adsorption [13].

Hydrophobic interactions are the main driving forces of protein adsorption on hydrophobic surface. On the other hand, in case of adsorption on hydrophilic and charged materials, electrostatic and van der Waals interactions take over as primary cause of protein binding to surfaces [20]. When a charged object is immersed into a solution, ions of opposite charges are attracted towards it, forming the so called double layer: the Stern layer, which is composed by ions very close to the surface, and the Gouy-Chapman diffuse layer, which extends towards the solution and exhibits an abundance of ions compensating the surface charge [23]. When proteins approach a surface, the protein diffuse layer overlaps with the one of the substrate and attractive or repulsive forces may arise depending on the zeta potential (ζ). The zeta potential is defined as the electrical potential at the interface dividing the Stern and the Gouy-Chapman layers. Proteins are also sensitive to dipole–dipole interactions, induced or not, commonly referred as van der Waals forces. Usually, van der Waals interactions are attractive at short distances [24]. The contribution of electrostatic interactions and van der Waals forces can be predicted by the DLVO (Derjaguin, Landau, Verwey Overbeek) theory. This theory allows to calculate the total interaction energy between two surfaces, determining if the resulting force is attractive or repulsive in the range of few tens of nanometers [24].

### 2.2. Surface Effect on Protein Adsorption

**Figure 1:**
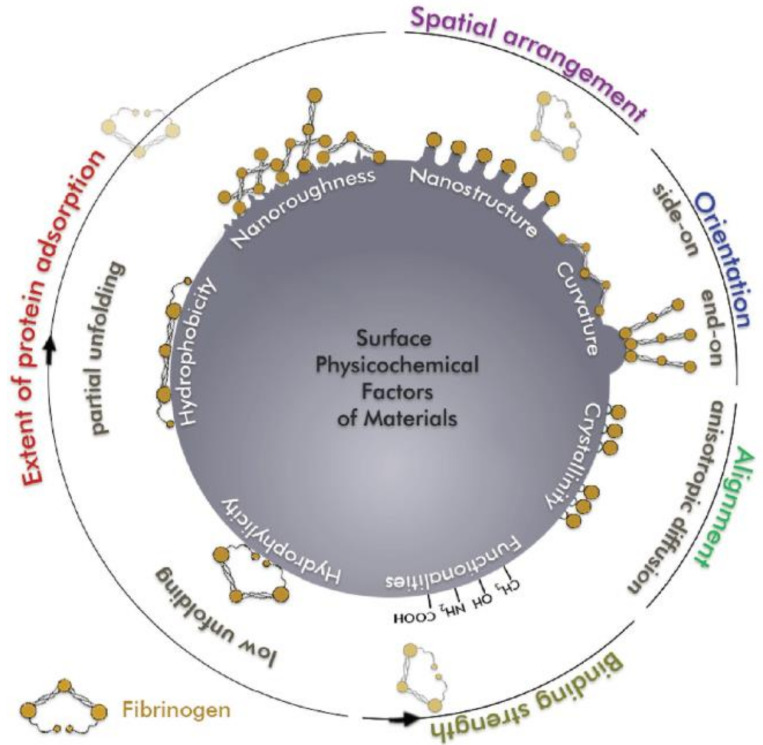
Effects of the physiochemical properties of material surfaces on various aspects of protein adsorption (amount, binding strength, orientation, conformation). Reprinted with permission from ref. [25]. Copyright 2017 WILEY-VCH Verlag GmbH & Co.

As discussed in the previous section, it is clear that the surface properties influence on protein adsorption mechanisms is of great importance. Surface wettability, topography, charge, and chemistry are able to deeply modify surface–protein interactions, as schematized in Figure 1 [12,25]. 

Wettability of substrates determines the major driving forces for adsorption, and the final result of the whole process may change heavily. It is well accepted that hydrophobic surfaces can adsorb more protein with respect to hydrophilic one [26]. Hydrophobic surfaces can better interact with hydrophobic residues of proteins and water displacement from the surface is more favorable than on hydrophilic surfaces [14]. The pivotal water contact angle (WCA)(θ) dividing hydrophobic from hydrophilic surfaces regarding protein adsorption was set at values near θ = 65° by different researchers [27,28]. 

Wettability does not only influence the total amount of proteins, which can be adsorbed onto surfaces, but also their spatial conformation. Different studies investigated the effect of surface wettability on protein conformation upon adsorption, such as FN and bovine serum albumin (BSA), highlighting the fact that proteins adsorbed on more hydrophobic materials present the higher degree of denaturation [29,30]. 

Strictly related to surface hydrophilicity/hydrophobicity behavior, there are surface charges. Usually, hydrophobic materials are non-polar, while hydrophilic substrates present a distribution of charges onto their surfaces. Surface charge of solids and particles immersed in solution may vary with the pH of the liquid, and they can be both positive and negative. Thus, according to protein type and substrate, attractive or repulsive interactions may happen [20]. Negatively charged surfaces, such as bioactive glasses in physiological conditions, may hinder adsorption of negative charged proteins [15], and vice versa for positive surfaces [31]. Still, proteins bearing an overall negative charge may adsorb on negatively charged surfaces thanks to the interactions of positive residues that remains on their surfaces, such in the case of BSA [32]. The same can happen with positive charges onto materials, as in the case of block co-polymers [33].

Cells are able to sense micro- and nano- roughness and it affects their adhesion, proliferation, and growth [8]. Similarly, morphological features of surfaces can influence protein adsorption [34]. Increase in surface roughness leads to larger surface area, thus higher protein adsorption. Still, it has been found that in some cases, proteins adsorb on appreciably larger amounts than what might be expected accounting only area increase [35]. Grain size and crystallinity of materials have also been reported to influence the adsorption process on biomaterials [36]

Protein adsorption can be controlled also by changing the chemistry of the surface, for example by functionalization with polymers or polyelectrolyte brush [12,36].

### 2.3. Protein Characteristics Affecting Adsorption

As well as surface characteristics, protein features like structural stability, charge, and dimension, are of greatly affect the adsorption mechanisms and outcomes. 

Structural stability of proteins is related to the easiness to undergo conformational modification. In the late 1990s, Norde introduced the concept of *soft* and *hard* proteins [9] to highlight the different structural behavior of proteins: Soft proteins are less stable and they are more prone to change their conformation after adsorption; on the other hand, hard proteins are more stable and they less prone to be denaturated. Changes in 3D conformation allows optimization of electrostatic interactions on hydrophilic/charged surface, since polar amino acid residues can be close to oppositely charged surfaces. Thus, in general, the soft proteins adsorb in a larger amount than the hard ones [37]. Changes in tertiary and secondary structure of proteins can affect their biological activity. In some cases, denaturation produces a loss of protein activity, as in the case of platelets activation by fibrinogen (FIB) [38]. In another case, denaturation can increase the effect of certain proteins. Denatured FN may expose more integrin-binding sites RGD, increasing cells adhesion [39].

Electrostatic interactions are capable to drive adsorption of proteins on charged surfaces of biomaterials, in a different way according to the overall net charge of proteins and distribution of the electrostatically charged residues within the protein’s structure. Proteins’ overall net charge is influenced by the presence of polar amino acids in the primary protein structure and by the pH of the solution, depending on the acid/basic behavior of the specific amino acids [40]. The overall net charge of a proteins is positive at pH values below the isoelectric point (IEP), zero at the IEP, and negative above it: It must be remembered that in any case the IEP is due to a balance between the number of positively and negatively charged functional groups which are present at any pH. IEPs of different proteins can vary in a wide range of values, from 4.7 of BSA to about 11 for Lys [41,42]. Still, proteins can adsorb to likely charged surfaces thanks to a non-homogeneous charge distribution within the protein’s structure: Positive residues can be selectively exposed on the outside of BSA even at pH 7, allowing its adsorption on a negative surface like titanium [32]. Furthermore, the proteins charges also determine the nature of protein–protein interactions. It is accepted that a higher amount of a protein is adsorbed at the IEP, when electrostatic repulsion between proteins is minimized [12]. Dimension and molecular weight of proteins play an important role too. The smallest proteins can reach the surface before the largest thanks to a higher diffusion coefficient, thus adsorbing in a larger amount at first. In addition, the largest proteins need to displace more water molecules, on hydrophilic surfaces, and higher energy is needed to enter the interface region [43]. On the other side, the largest proteins usually can interact in a stronger manner with the surface, therefore they can displace and substitute the smallest proteins with times. This dynamic behavior of the adsorbed protein layer is known as “the Vroman effect” [12].

### 2.4. External Parameters Affecting Protein Adsorption

Investigating protein adsorption, it is also fundamental to consider the environment where the process is carried on and the effects of some external parameters. Temperature influences several aspects of the adsorption mechanisms. Increasing temperature allows for higher mobility of proteins in solution, faster adsorption kinetics [44], equilibrium surface concentration [45], and also protein desorption from the surface is easier [29]. 

A fundamental and determining role is played by the solution where proteins are dissolved. As already mentioned, the pH determines both the charge of protein and surface. Thus, according to its value, the same protein–surface combination may repel or attract one another [46]. Beside pH, other solution parameters have great influence on the adsorption. Presence of ions in the solution has a double effect. The first one regards the ionic strength of the solution, which influences the thickness of the Gouy-Chapman layer and consequently the distance at which the electrostatic interactions take place. Higher ionic concentration and strength result in a thinner diffuse layer both on proteins and surfaces. Thus, an eventual electrostatic repulsion is less relevant on adsorption [47]. As a second effect, ions can also compete with proteins in the adsorption on surfaces. For example, negative phosphate ions in phosphate buffered saline (PBS) solution have been found to depress protein adsorption [48].

Protein concentration in solution is another parameter that can largely impact on adsorption. Several studies describe how an increase of the initial protein concentration results in a higher amount of protein adsorbed onto the surface at the equilibrium [49,50].

The main parameters that affect protein adsorption and the general rules of thumb on the process are collected in Table 1.

## 3. How the Characteristics of Titanium Based Biomaterials Influence Protein Adsorption

### 3.1. General Consideration on Protein Adsorption on Titanium Based Materials

**Figure 2:**
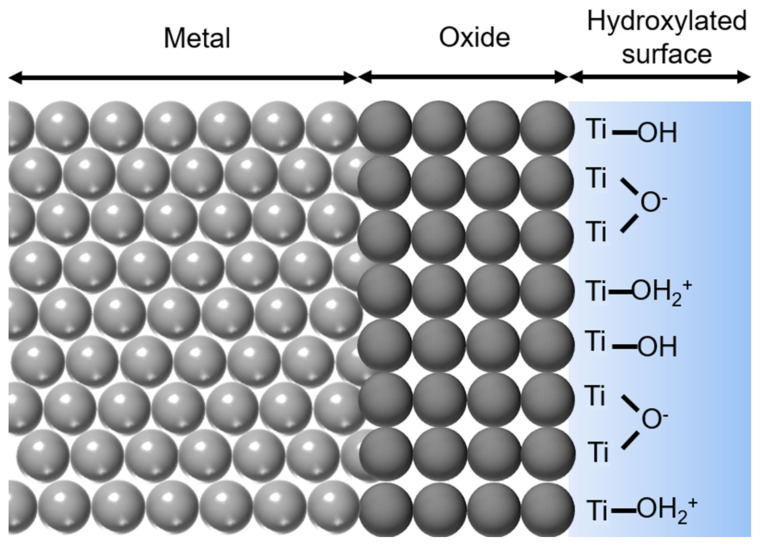
Scheme of hydroxylation of Ti surface and surface charge generation during contact with aqueous solutions.

Titanium peculiar properties have made it one of the most world widespread biomaterials [1]. With respect to other metals, titanium and its alloys possess excellent osseointegration capability, proper mechanical properties, and soft tissue compatibility. They have been extensively described elsewhere, therefore here only the ones of interest for discussing protein adsorption will be briefly reported [8,51,52]. Being a very reactive element, titanium does not exist in its metal form onto its surface, but it is immediately passivated by oxygen and its surface is covered by a thin native oxide layer, which is mainly amorphous TiO_2_ about 3–7 nm thick [53]. This layer confers chemical stability, biological inertness and corrosion resistance to the surface. To understand how this biomaterial interacts with the biological environments, it is mandatory to notice that titanium surfaces are highly hydroxylated. OH groups can form by dissociation of water molecules at the five-coordinated Ti sites. Several kinds of hydroxyls can thus form on the surface, differing in their position (terminal or bridging) and in their chemical behavior (acidic or basic). As a consequence, when the surface gets in contact with water, acidic OH groups deprotonate, while basic OH groups protonate themselves, forming both positive and negative charges (Figure 2).

Acidic OH deprotonation: Ti-OH + H_2_O ↔ [Ti-O]^−^ + H_3_O^+^

Basic OH protonation: Ti-OH + H_2_O ↔ [Ti-OH]^+^ + OH^−^

As consequence of the dissociation constants of both the OH groups, IEP of titanium lies around 5 [54].

Titanium affinity for proteins is well acknowledged. A stable protein layer was found to form in vivo after just three hours [55]. This was observed to prevent precipitation of compound like HA after a week. Ti-based materials can interact with many different proteins, such as serum albumin [56,57], FIB [58], or FN [59]. Many researchers have tried to unveil the mechanisms of interaction between proteins and titanium substrates. By studying the adsorption isotherms of several different proteins (such as human serum albumin (HSA), BSA, lysozyme (LYS), pepsin, myoglobin, and others) at different pH values, Imamura et al. [60] ascribed pseudo-irreversible adsorption of protein to electrostatic interactions between the OH_2_^+^ groups on the titanium surface and COO^-^ groups of proteins. Furthermore, negatively charged carboxyl groups can also induce protonation of the OH groups of the surface around the IEP of titanium. Another research group proposed a slightly different interaction mechanism between HSA and titanium [57,61]. They proposed that HSA has a similar effect to a local change of the pH, acting like a reduction of [H^+^] and affecting the thickness of the H^+^ diffusion layer on the Ti surface. Interactions between albumin and a titanium surface followed the proposed two steps mechanism, which involves a first hydrogen bonding and a subsequent proton transfer.
Hydrogen bonding:
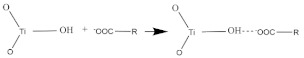
Proton transfer:
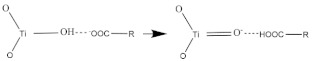


Even though the interaction mechanisms proposed by Imamura [60] and Camàra [57] are different, they underline the importance of the OH groups on driving protein adsorption. Molecular dynamic simulations observed that an increased density of the OH groups on rutile (1 1 0) means higher affinity for the subdomain IIIb of HSA [62]. Electrostatic interactions with the COO^-^ and NH_3_^+^ groups of proteins were greatly enhanced. A key role of the local electrostatic interactions between opposite charges respectively on TiO_2_ and organic molecules was also observed for peptides [63]. The charge effect of hydroxyls on the strength of protein adsorption was investigated in another interesting computational studies by Sun et al. [64]. They tuned the hydrophobicity/hydrophilicity of rutile surface by scaling the OH charges of different factors. Lower surface charge, related to higher hydrophobicity, turned out to adsorb lactoferrin and bone morphogenetic protein-2 (BMP-2) in a stronger manner than a hydrophilic surface. Being a soft protein, BMP-2 is also more denaturated. At the same time, protein–surface interactions on hydrophilic TiO_2_ surfaces are hindered due to water-surface interactions [65]. Thus, spreading and denaturation of certain adsorbed proteins are limited. OH groups generated onto TiO_2_ by vacuum annealing can prevent FIB denaturation by avoiding electron transfer, from the protein to the surface. In this case, hydrophilic TiO_2_ surface denatures less proteins than the hydrophobic ones [38]. Less FIB denaturation is related to lower platelet activity and better blood compatibility of biomaterials.

In order to predict the biological behavior of biomaterials, alongside the amount and type of protein adsorbed, it is necessary to be aware of their spatial configuration and orientation with respect to the surface. Proteins can adsorb in a “side-on” or “end-on” orientation, according to the positioning of their main axis [12]. 

Furthermore, denaturation can occur to different extents with different proteins. As already mentioned, adsorption on titanium substrates lead to denaturation of FIB. Several authors found that FIB can interact with titanium surfaces through αC domains. Since they are positively charged at pH = 7.4, electrostatic attraction between the surface and the protein can occur [58,66]. The strong protein–surface interactions lead to denaturation of FIB [66]. This was confirmed by Zhao et al. [67]. Furthermore, even though binding via αC domains shall result in side-on orientation of the proteins on the surface, FIB was found with preferred end-on orientation [67]. Bimodal adsorption isotherms of immunoglobulin (IgG) suggest that adsorbed proteins may undergo structural rearrangements and orientation modification according to saturation level of the surface [68]. While in some cases no denaturation of BSA was observed upon adsorption on titanium oxide [69], others had observed conformational changes of albumin [56]. Hydrophobicity of titanium may lead to spreading of adsorbed HSA onto its surface [70]. Conformation and adsorption mechanisms are strictly dependent on both surface features and protein composition. Different structures were found for proteins that shall be analogous, such as chicken and human albumin [71]. Adsorption mechanism was also profoundly different: HSA adsorbs as a continuous thin film, while chicken albumin forms adherent flakes on the titanium surface. In addition, relevant peptide sequences, such as RGD domains of FN, may change their spatial configuration after adsorption on rutile or anatase [72] 

**Figure 3:**
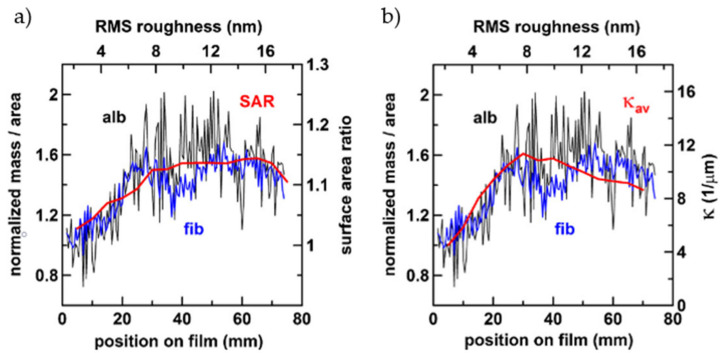
Normalized adsorption profile of bovine serum albumin (BSA) (black) and fibrinogen (FIB) (blue) on Ti with roughness gradient (left y-axes). The overlaid red lines are the SAR profile (**a**) and the curvature profile (**b**) (right y-axes). Adapted with permission from ref. [76]. Copyright 2011 Elsevier B.V.

Surface roughness is a parameter that very often is addressed as pivotal in determining the outcome of protein adsorption, and more in general, cell behavior [73]. It does not only change the effective surface area available for interaction with proteins, but it can also affect the wettability of the materials. It has been acknowledged that roughness in the micro-range enhanced protein adsorption due to increase of specific surface area [74]. Several authors have tried to understand the extent of roughness, in particular in the nano-range, influence on the protein adsorption on titanium-based surfaces. Roughness variations from few to some tens of nm were found not to have a unique effect on all proteins and some results in literature may disagree. BSA adsorption is slightly influenced by roughness between R_a_ values of 1.57 and 16.44, while in the same range FIB adsorption is increased to a slightly larger extend [75]. Controversially, in a more recent study, Rockwell et al. [76] observed that the increment in the surface area ratio (SAR) due to increased roughness, in the same range as previously reported [75], along sample profile, was not sufficient for explain the increased of normalized adsorption of both FIB and BSA (Figure 3a). Increments on proteins and SAR were up to 50% and 15%, respectively. Instead, the increment in curvature of surface features accounted better for the increment in adsorption (Figure 3b): Higher curvature, meaning smaller features radii, favors end-on FIB adsorption and stabilizes protein secondary structures. Besides, increased roughness, from less than 1 nanometer to about 11, resulted in increased surface free energy (SFE) that promoted better adsorption of FN and VN from fetal bovine serum (FBS) [77]. These results were confirmed also on TiO_2_ when other proteins, such as BSA [78] or casein [79], were adsorbed.

Roughness of titanium substrates is also capable of influencing the mechanisms of adsorption. While adsorption of BSA, FIB, and streptavidin on flatter substrates occurs mainly as protein monolayers, roughness values about R_ms_ = 29.5 nm can increase protein–protein interactions, resulting in a multilayer type adsorption [80]. Surface features such as protuberances and peaks are not the only topographical characteristics that have influence on proteins adsorption. Surface pores in the meso- and nano-range need to be accounted when the effect of surface roughness on this matter is discussed. Proteins are not able to enter pores smaller than their hydrodynamic radius. In the case of BSA, of which hydrodynamic diameter is about 7.2 nm, mesopores need to be at least about 9 nm for albumin to enter them [81]. Larger mesopores can accommodate more than one BSA molecule, with very little conformational changes, and protein-surface adhesion forces were stronger with respect to smaller pores [82]. Singh et al. [83], due to protein tendency to aggregate into nanopores, concluded that nanometer scale morphology is the main reason for increased protein adsorption, more than the modest increase in wettability of surface with different roughness. 

Titanium and its oxide exhibit different adsorption properties with respect to other materials, such as other metals or metal oxides clinically used polymers or dental enamel. In comparison with other metal surfaces such as Au, Pt, and Ir, titanium adsorbs the largest amount of plasma proteins. This is because Ti presents the highest SFE and roughness, as a result of the deposition process of metal thin films [84]. At the opposite, when TiO_2_ is compared with other oxides such as ZrO_x_, TaO_x_, and NbO_x_, it showed the least adsorption capability [85] and it is also the flattest and the least energetic surface. ZrO_x_ interacts the most with albumin being a hydrophobic surface, while the amount of adsorbed BSA correlates well with roughness and polar component of the SFE on the hydrophilic oxides. Similar evidence of different mechanisms of adsorption on hydrophobic or hydrophilic surfaces was found with FIB, investigating fibrinogen adsorption on the same set of oxides [86]. Titanium shows also different retention capability of the adsorbed proteins with respect to the other oxides. Its negative charge makes HSA displacement from its surface faster than on positive charged alumina, at pH 7 [87]. Al_2_O_3_ adsorbs more BSA than TiO_2_ also because of its higher number of OH groups that can form H-bonds with proteins [88]. Adsorption of positively charged proteins such as lactoferrin was enhanced on titanium with respect to stainless steel, ZrO_2_, and polymethylmethacrylate (PMMA) thanks to the higher negative surface charge [89]. Still, stronger interactions were found on hydrophobic substrates. In order to better understand why different materials have different behavior during their life as implants, titanium was widely compared to other surfaces of interest in the dental field. Titanium’s poor adhesion to gingival tissue may be explained by the fact that, with respect to dentin, it adsorbs less key basal lamina proteins, such as laminin (LAM) α, a protein with a key role in tooth-epithelium adhesion, and nidogen-1 [90]. It was also observed that hydrophobic polymeric materials used in dental field, such as polytetrafluoroethylene (PTFE), polyethylene (PE), and PMMA, adsorb more salivary proteins than Ti, for instance salivary mucins and proline-rich proteins [91,92]. This also reflects in higher adhesion forces between albumin and polymers as PMMA and PTFE with respect to titanium [93]. Interestingly, the interaction force between BSA and Ti is about twofold more than on enamel.

### 3.2. Effect of Surface Modifications on Titanium: How Topography, Roughness and Surface Chemistry Change Protein Adsorption

#### 3.2.1. Surface Modification by Sand Blasting and Acid Etching (SLA)

Surface roughness and wettability are the main parameters influenced by SLA treatments, thus changes in protein adsorption are mainly ascribed to these materials features. According to our findings in literature, the studies on this kind of surfaces are not in complete agreement. Some of them observed that SLA treatments increases the total amount of adsorbed proteins [94,95], while, in different conditions of adsorption, others observed a neglectable difference [96]. In a remarkable work of Kohavi et al. [94], the authors studied the influence of SLA and acid treatments on Ti64. Proteins adsorption was carried out in vivo during dental implantation surgery. A titanium rod was implanted into the osteotomy and removed after 10 min. Albumin, fibronectin, fibrinogen, and immunoglobulin were quantified by enzyme-linked immunosorbent assay (ELISA). The SLA surfaces adsorbed more than fourfold more of each protein with respect to an untreated surface. Acid etched (AE) titanium surfaces adsorbed only twice more. SLA surfaces were rougher than both AE and flat surfaces (R_a_ equal to 287.5, 214.5 and 26.8 nm, respectively). Roughness was addressed as the main factor influencing in vivo protein uptake. Prewetting of surfaces also increases protein adsorption. Similar findings on the same surfaces were obtained in vitro [95]. FN resulted the major protein found on a surface in case of adsorption both from a single protein solution and whole plasma. The effect of roughness and increased surface area on protein adsorption was also highlighted by SLA treatment followed by secondary etching [97]. Protein adsorption is only increased to a certain time of etching, since after about 30 min decreasing in specific surface area is experienced. MC3T3 pre-osteoblastic cells viability also correlate well with this observation. Kopf and co-workers [74] put effort in isolate the effect of wettability and roughness. Hydrophilic and hydrophobic SLA surfaces were obtained through proper storage in air or NaCl solutions. On some samples, the storage in NaCl resulted in further nanostructuration of the surface. WCA of hydrophilic and hydrophobic SLA surfaces ranged between less than 10° to 120°, respectively. Simply, SLA-treated surfaces showed no influence of the WCA on adsorption of both FIB and FN. On the contrary, the hydrophilic nanostructured (NS)-SLA samples adsorbed much more than the hydrophobic ones. In both cases, they adsorbed more than the SLA specimens. Thus, it seems that protein adsorption is mainly driven by roughness at the microscale and by a synergistic effect of hydrophilicity and roughness when it comes to nanostrcutures. As an interesting fact, in the same study, it is observed that blood clotting is more improved by hydrophilicity than surface topography. H_2_O_2_ hydrothermal treatments on SLA-treated dentals screws can promote bioactivity through surface nanostructuration and formation of many OH groups [98]. Better protein adsorption, in particular increased selectivity towards FN, resulted from increased wettability of the implants. Hydroxylation of the surface does not only account for improved wettability and enhanced protein adsorption. SLA-induced OH groups are also responsible for denaturation of proteins, such as statherin [99]. Hydroxyls can bond with proteins through hydrogen bonds, disrupting the equilibrium of forces that maintains the native conformation of proteins. Statherin adsorbed onto polished titanium showed less denaturation.

**Figure 4:**
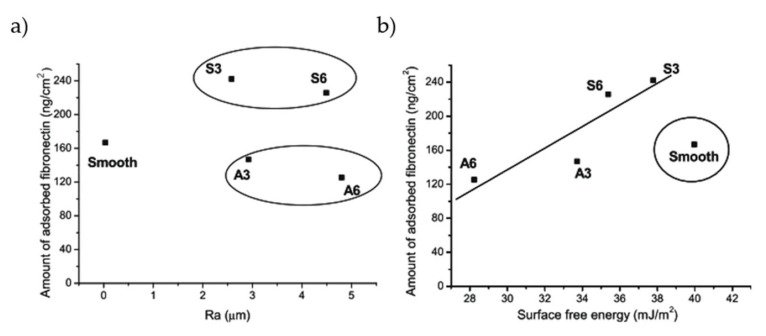
Correlation of FN adsorption with roughness (**a**) and surface free energy (SFE) (**b**) on cp-Ti blasted with different particles: S, SiC particles; A, Al_2_O_3_ particels; 3, particles of 212–300 µm; 6, 425–600 µm. Adapted with permission from ref. [100]. Copyright 2009 Acta Materialia Inc. Published by Elsevier Ltd.

Some studies focused on how SFE influences the adsorption of proteins. Simple sandblasting of cp-Ti resulted in very different values of surface energy, according to dimension of the blasting particles and even to their composition [100]. On rough surfaces, the authors found a linear correlation between surface energy and amount of adsorbed FN (Figure 4b). Interestingly, the samples treated with SiC particles showed higher SFE, in particular the dispersive component, than the ones processed with alumina particles. As a consequence, FN adsorbed preferably on SiC-blasted samples, regardless of roughness (Figure 4a). The importance of SFE on SLA treatments was also observed very recently by Mussano et al. [101]. Adsorption of different proteins, namely collagen (COL) I, FN, and BSA, was depressed by blasting with alumina if compared with machined surface. SLA treatment restored titanium adsorptive properties, though without enhancement with respect to untreated surface. Blasted surfaces showed lower SFE, while machined and SLA specimens had similar values. This correlates well with the results obtained for alumina blasting particles in ref. [100].

As previously said, the debate on the effective enhancement of protein adsorption by SLA treatments is an open issue. A recent study observed no difference in adsorption from FBS on machined or SLA cp-Ti [96]. Still, microrough surfaces elicit murine MC3T3-E1 osteoblastic cell spreading and adhesion. Similar findings were observed also in adsorption kinetics and total amount of adsorbed proteins when FN and BSA are adsorbed from single protein solutions, even when wettability was increased by heat treatment [102]. As an interesting fact, heat treatment promoted selective adsorption of fibrinogen and fibronectin from human serum. Even though hydrophilicity may not increase protein adsorption on SLA surfaces, it can promote the formation of a more homogeneous protein layer [103]. SLA did not seem to enhance adsorption of salivary proteins neither [104]. 

#### 3.2.2. Surface Modification by Chemical and Hydrothermal Treatments

Acid etching is a very simple kind of chemical treatment employed to enhance biological response of titanium surfaces [51]. Nanopatterning by acid etching was found to affect in different ways adsorption of different proteins [105]. Nanopits, generated by simultaneous acid etching and oxidation with H_2_O_2_, act as physical traps for proteins that can be accommodated within, such as LYS and growth/differentiation factor 5. Adsorption of larger proteins, such as FN, is hindered due to steric limitations. Acid etching of microgrooved titanium resulted in increased hydrophilicity and consequent enhancement of BSA adsorption and human osteoblast proliferation [106]. 

Hydrothermal treatments are widespread techniques to obtain surfaces with enhanced cytocompatibility [51]. Immersion in solutions with different chemicals and subsequent heating results in nanostructuration of the surface and modification of its chemistry.

Hydrothermal treatments on Ti64 can also be obtained using simply distilled water [107]. In this way, higher hydrophilicity is obtained without changing surface roughness. Increased wettability led to higher laminin adsorption and consequent improved adhesion of cells through integrins. Hydrogen peroxide is a common reactant for hydrothermal modification of titanium surfaces. Nanoporous structures can be obtained in this way [108]. The increased roughness and SFE of H_2_O_2_-treated Ti64 results in evident decrease of the WCA, from 49° to 16°, and in a sixfold increase of cytochrome C adsorption. Enhanced serum protein adsorption on this kind of surfaces is also due to the generation of OH groups on titanium surfaces [109]. BSA adsorbs also in a different conformation on H_2_O_2_-treated Ti64 with respect to the polished surfaces [54]. The higher amount of OH on the treated surface produced adsorption of albumin in a more hydrophilic orientation. FN was proven to adsorb in an island-like manner on this kind of surfaces, by positioning mainly in the surface valleys and forming multilayered globular structures ranging from 55 to 83 nm in diameters [110]. Titanium oxide grown using H_2_O_2_ treatment adsorbs FN in a more irreversible manner than sputtered TiO_2_. On the other side, adsorbed HSA is more easily exchanged by HSA molecules in solution [111].

**Figure 5:**
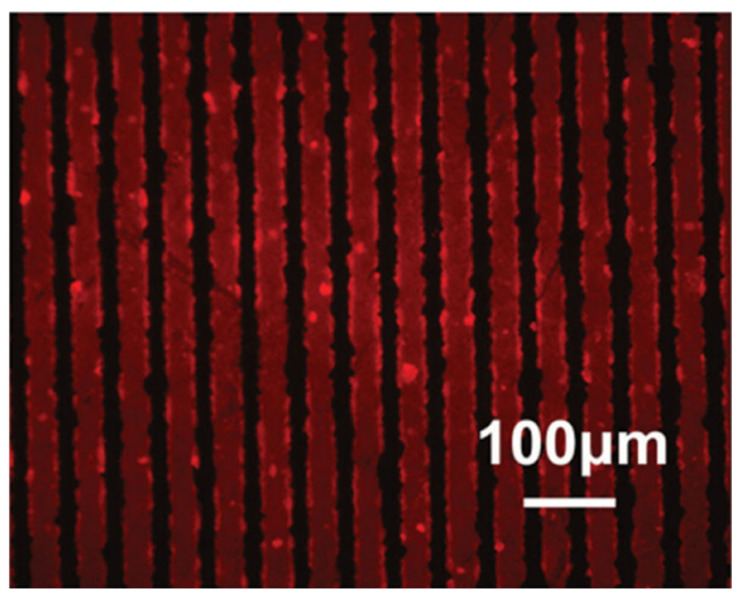
Fluorescent image of BSA adsorbed onto a patterned nanostructured surface: The protein is adsorbed on zones with titanium nanoneedles (red) and not in the zones, which were irradiated with laser. Adapted with permission from ref. [119]. Copyright 2015 WILEY-VCH Verlag GmbH & Co. KGaA, Weinheim.

Bioactive titanium surfaces can be obtained by acid-alkali (AA) treatments, which involve a step of acid etching and a subsequent treatment in alkali solution, mainly NaOH. Both the steps can be performed at temperatures ranging from 30 °C [112] up to 70 °C [113]. These treatments allow to obtain surfaces with nanostructures, enhanced wettability, and different charges with respect to untreated titanium. Nanoscale topography was found responsible for increased protein adsorption of albumin and fibronectin in particular [112]. Treatments in NaOH result in a formation of Ti-O-Na layer that changes the surface electrical charge further increasing adsorption of negatively charged proteins such as albumin. AA treatments are more effective in promoting protein adsorption when compared to other surface treatments, such as alkali-heat (AH) [114] or anodic oxidation treatments (AO) [115] and also SLA modification [116]. Better BSA adsorption capability of AA-Ti than AH-Ti, where samples are heated at 600 °C for 1 h after alkali treatment, relied on the higher number of OH groups and on the positive surface charge of AA-Ti [114]. Hu and Yang observed that the NH_3_^+^ groups of albumin mainly interact with AA and untreated samples, exposing more COO^-^ groups while the orientation is different on AH-Ti. Secondary structures of albumin are also affected by the charge of the surfaces and OH groups. Interestingly, they found that BSA preadsorption elicited higher mouse osteoblast proliferation on polished Ti (P-Ti), due to higher content of cell binding α-helices. The same research group observed that AA-Ti adsorbs more osseointegration-relevant proteins, such as FN and bone morphogenetic protein 2 (BMP-2) than AO- and P-Ti [113,115]. They observed that morphology was more relevant than wettability in determining the amount of protein adsorbed: Nanopits on AO-Ti are not able to accommodate large proteins, while grooves on P-Ti and network structure of AA-Ti offers more interaction sites. The latter can act as reservoir for BMP-2 [113]. On the other hand, protein conformation on the surface is dictated by hydroxylation of surfaces. Thus, proteins retained their native structure better on AO-Ti than on AA-Ti. Biological activity of BMP-2 is related to its α-helix content, thus AO-Ti promoted bone formation to a longer extend than AA-Ti, despite adsorbing less. Contrary, adhesive properties of FN are more related to β-sheets, which are consistent with the amount of RGD sequences. In this case, AA-Ti can increase FN effect thanks to the disruption of α-helices and the formation of β-sheets [115]. AA-treatments were also found to increase protein adsorption of SLA modified surfaces by turning the surface from hydrophobic to super-hydrophilic [116]. On hydrophobic samples, air bubbles may be trapped in micropores in a Cassie-Baxter regime, hindering solution-surface interactions thus reducing protein adsorption. Moreover, AA-treatments increase SFE. Alkali-acid treatments were employed also to increase protein adsorption ability of porous titanium scaffolds [117,118]. Various morphology can be easily obtained by changing the treatment parameters such as temperature, time, and solution compositions. Nanoneedles [119], nanopores, or nanoleaves [120] can be obtained on the surfaces. Since nanoneedles showed much higher BSA adsorption than the untreated surfaces, Yu et al. [119] obtained very specific adsorption patterns by texturing nanostructured titanium with laser irradiation (Figure 5). 

Morphology has also effect on adsorption selectivity. They present different surface area ratios, SFE, or they can generate physical nanotraps for proteins of certain dimensions [120]. Thus, nanoneedles may overall adsorb less proteins from FBS than nanoleaf or octahedral structures, but still have an equal if not higher number of adhesive proteins such as FN and VN. As result, focal adhesion of human osteoblasts turned out to be larger on this kind of nanostructured surface than on others. Depending on the kind of protein, adsorption may be mainly driven by the contact angle or by roughness. In the case of FIB, adsorption on hydrothermally treated cp-Ti and Ti64 resulted affected more by topography than WCA [121]. Protein adsorption on different morphologies obtained by hydrothermal treatments was also related to their surface potential [122]. High treating temperature, 140 °C, allows to obtain nano-wires on the surface, which exhibit the lowest zeta potential, about −50 mV at pH 7.4, among other nano-structures, such as a nano-network or nano-plate, about −30 and −35 mV at pH 7.4, respectively. Adsorption of BSA and FN was higher on nanowires than on all the other surfaces. Moreover, mouse bone marrow MSCs (BMSCs) had spread better on this kind of surface. 

#### 3.2.3. Growth of Titania Nanotubes (TNTs)

A common and easy way to obtain nanotextured titanium surfaces is formation of nanotubes by anodic oxidation [8]. TNTs geometrical features such as diameters, in the range of 15–300 nm, and length are easily tunable with the process parameters. Such surfaces have higher biological response than untreated Ti and can induce cellular differentiation. 

**Figure 6:**
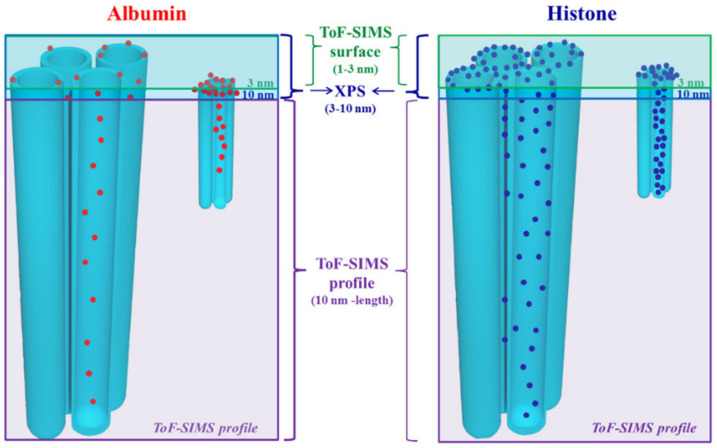
Spatial distribution of albumin and histone adsorbed on titania nanotubes reconstructed by different techniques: Time of flight-secondary ion mass spectroscopy (ToF-SIMS) (1–3 nm depth); X-ray photoelectron spectroscopy (XPS) (3–10 nm depth); Tof-SIMS depth profile (from 10 nm to bottom). Reprinted with permission from ref. [128]. Copyright 2016 Acta Materialia Inc. Published by Elsevier Ltd. Amsterdam, The Netherlands.

At first glance, the enhancement of protein adsorption on TNTs can be ascribed to a much larger surface area than a flat sample [123,124]. At the same time, the oxidized surfaces have higher wettability and SFE, which are factors that contribute to BSA adsorption. The diameters of TNTs further influence the amount of protein adsorbed [125,126]. Increasing diameters from 30 to 100 nm increase adsorption of both FN and COL [125]. Osteoblast viability is higher on 30 nm TNTs when no proteins are adsorbed, while they have the same viability on 30 and 100 nm tubes after adsorption of FN and COL. Computational studies showed that larger diameters correspond to higher interaction energy with collagen, thus increasing protein adsorption [127]. Conformation of the proteins is not affected by TNT’s diameters, and collagen lies across several nanotubes. Changes in the 3D structure of other proteins were reported. Smaller diameters correspond to higher α-helix and β-turn content of adsorbed BSA and FIB, while β-sheet showed the inverse behavior. Since the bigger the diameter, the larger flat area on the top of TNTs is, conformation is similar to the proteins adsorbed onto a flat surface. With smaller nanotubes, proteins are more likely to interact with the edge of them, generating differences [126]. A very interesting study by Kulkarni and co-workers [128] defines the synergistic effect of dimensions and charge distribution of TNTs on protein adsorption. The surface charge density is affected by radius of curvature, therefore there is a difference between the outer convex surface and the inner concave surface of TNT. The former presents higher density than the latter. Anyway, the points with higher curvature are the edges at the top of TNTs. Small proteins like histone and albumin can enter TNT with diameter ranging from 15 to 100 nm. Being positively charged, histone can adsorb twofold BSA and also penetrate the space between nanotubes. Albumin cannot do that because of electrostatic repulsion with titanium oxide. Edges at the top of nanotubes are preferential adsorption sites for histone due to higher charge density, as shown in Figure 6

Adsorption from platelet rich plasma resulted in lower FIB on TNT surfaces with respect to flat cp-Ti [129]. This is because on more hydrophilic surfaces, such as nanotubes, fibrinogen can be more easily replaced by other proteins, like kininogen, through “the Vroman effect”. Adsorption of proteins can be selectively controlled by tuning the diameters of the tube. Smaller TNTs, about 27 nm of diameter, adsorbed more VN from FBS than larger ones, diameters of about 88 nm [130]. Similar effect was obtained for other adhesive proteins such as laminin and fibronectin. Protein adsorption on TNT can be further enhanced by chemical modification of the surface. Hydrogenation of the surface can be achieved by thermal treatments in hydrogenated atmosphere [131]. This is because hydrogenation increase hydrophilicity of TNT and liquid penetration as consequence. Since TNTs substrates can be used as drug-carrier materials, hydrogenation treatment is intriguing because of its effect in changing the release profile of the different proteins. 

#### 3.2.4. Other Surface Modification Techniques

SLA, chemical treatments, and growth of TNTs are the most common surface modification techniques for Ti-based biomaterials. Beside those, studies regarding how other kind of surface modifications affect protein adsorption were found. 

Electrochemical methods such anodic oxidation allows to grow oxide layers with different nanostrucutres: nanopores [132], nanonetworks [133], or nanorods [134]. By increasing the applied voltage, thickness, micro-roughness, and porosity of the oxide layer increase, resulting in higher BSA adsorption [132]. Higher anodizing voltages resulted, on the other hand, in a rutile layer, which is less biocompatible than anatase. On similar surfaces, no enhancement of adsorbed protein was found when high protein concertation solution as FBS was used [135]. This is a useful reminder that protein adsorption on surfaces is not only dictated by the biomaterials properties but also, and in a significant manner, by the adsorption environment. Subsequent hydrothermal modification of the anodized surfaces highlighted the effect of the surface charge on protein adsorption [136]. Anatase nano-spikes lowered the surface potential of titanium and showed inhomogeneous charge distribution (higher negative charge density on titania tips due to higher curvature). Thus, adsorption of positively charged histone was increased. Au and Ag- nanoparticles (NPs) were successfully embedded into the titanium oxide layer by sequential anodization and soaking in NPs precursor solution and it was found that their presence further increases BSA adsorption [137]. Increased adsorption capability of surfaces with nanonetwork porosity was addressed as result of increased surface area, where pores can easily accommodate BSA and FN [133]. On nanorods, adsorption was found to be mainly driven by the density of the rods. When there are too many or too less structures, adsorption was found to be lower than on untreated titanium. Only intermediate rod density was beneficial for protein adsorption, MC3T3-E1 cell proliferation, and bone formation in vivo [134].

Surface texturing with laser beam is a rather novel way of obtain specific surface pattern in order to increase biological response to biomaterials [138]. It is possible to obtain very complex surface structures, such as micro-pits with nano-ripples at the bottom or at the top, selectively. This results in an accelerated adhesion of MSCs and in a more enhanced osteogenic behavior of the cells [139]. Laser patterning changes surface properties, such as roughness, wettability, chemistry, and charge, to a great extent. Thus, its effect on protein adsorption may vary largely according to the process parameters. Patterning of Ti64 was demonstrated to increase FIB adsorption due to increasing in surface roughness [140]. Furthermore, affinity for FN seemed increased, in particular due to increase in the polar component of the SFE [141]. For the same reason, adsorption of HSA decreased. Lower affinity of textured Ti64 for albumin was also ascribed to a reduction of available binding sites and to chemical modifications and formation of less active titanium oxide forms [142]. Controversy, in a series of studies by Kuczyńska et al. [138,143], an increased adsorption of both BSA and FN was observed. This is the combined result of modified wettability and SFE, morphology, and increased negative charge of the treated surfaces. Conformation of proteins was also affected.

### 3.3. Effect of Alloying Elements and Surface Ion Doping

Despite being the most widespread materials for orthopedic and dental implants, properties of pure titanium and Ti6Al4V alloy, such as Young’s modulus, are not the optimum for instance to avoid stress shield effect. Thus, titanium alloying with several different metals have been developed in order to reduce the elastic modulus or to get other interesting mechanical properties. Nickel is one of the most common alloying elements, TiNi alloys, such as Nitinol (about 50% Ti 50% Ni), possess shape memory and super-elastic properties. Nitinol is largely used in the manufacturing of vascular stent, for example [144].

Alloying elements not only modify the bulk properties of titanium, but also the surface ones. This affect adsorption of proteins. Higher Ni content, from 49.5% to 50.5%, in TiNi alloy results in lower albumin adsorption (from about 90 to 30 ng/cm^2^), while FN is quite unaffected. Both resulted in being largely lower than on cp-Ti, twofold and almost 4 times, respectively. Albumin adsorption was found to be proportionally related to the polar component of surface energy, and Ni can reduce it. Fibronectin is more affected by other factors, such as surface charge [145]. Regarding albumin, different results were obtained by Clarke at al. [144] by modifying the composition of the oxide layer on TiNi alloy. They obtained higher adsorption with higher Ni and lower O content in the oxide, regardless of contact angle and roughness. According to Bai studies with binary alloy of Ti with Cr, Al, or Ni oxide layer composition has a larger control on protein adsorption than the bulk ones [146]. In addition, FIB was found to be adsorbed less on Nitinol than on cp-Ti [147]. In both cases, it adsorbs with a “side-on” orientation. FN was found to adsorb in similar manner on cp-Ti also when Zr is introduced into TiNi alloy [148]. 

Niobium is another very common alloying element for titanium. Nb lowers the Young’s modulus of titanium, getting closer to the bone value [149]. β-alloys Ti-Nb-Zr and Ti-Zr showed very little differences in BSA adsorption with cp-Ti and Ti64, but a slight increase can be observed thanks to higher Zr content [150]. The oxide layer drives interactions with proteins also for this kind of alloys. In fact, introduction of boron ions causes a reduction of oxide thickness and hydroxide groups, hindering adsorption of proteins from FBS. This is also detrimental for MG63 human osteosarcoma cells proliferation [151]. Niobium is a beneficial element for proteins also when it is introduced into more complex alloys. The Ti-Zr-Pd-Si-Nb alloy showed enhanced adsorption of BSA and FN with respect to the Nb-free alloy thanks to improved hydrophilicity [152]. The importance of non-polar component in the adsorption of FN was highlighted by Herranz-Diez et al. [153]. They observed that very different Ti-based materials, namely cp-Ti, Ti64, and Ti25Nb21Hf, adsorbed very similar amount of fibronectin, despite various contact angels. Analyzing the components of SFE, they notice different values in the total SFE and polar component, cp-Ti showed the lowest ones. Instead, no variations were found in the dispersive components.

Several metallic ions are well known for being able to stimulate different biological responses, particularly in the field of bioactive glasses. As an example, Ca^2+^ favors osteoblast proliferation and differentiation, Zn^2+^ possess anti-bacterial and anti-inflammatory properties, and Mg^2+^ increase bone cell adhesion and new bone formation [154]. Thus, surface treatments of titanium materials have been developed over past years in order to introduce different ions, in particular within the oxide layer [8]. Presence of ions in the surface results in changes of biomaterials physio-chemical properties and, obviously, this affects protein adsorption. According to several authors, enhancement of protein adsorption is due to increased surface charge of ion-doped titanium materials [31,155] or the bridging effect of divalent ions [155]. Some of the most common methods to produce ion-containing titanium surfaces are hydrothermal treatments [155,156,157,158], which allows to obtain at the same time a nanostructured surface and ionic doping. Higher adsorption of albumin was found on treated cp-Ti with Mg^2+^ or Ca^2+^ ions with respect to Na^+^. Additionally, increased adsorption was obtained by increasing ions concentration [155]. Magnesium bridging effect towards protein was confirmed in other studies [157] and treated titanium turned out to be bioactive, inducing hydroxyapatite precipitation, and promoting osteoblast attachment and spreading [159]. Anyway, cell adhesion can be depressed by too high Mg concentration in the TiO_2_ layer due to much higher content of BSA, which reduces cell focal adhesion [160]. Similar results were obtained by lithium ions [31]. Treated surfaces showed super-hydrophilicity, ascribed to increase in surface energy, charge, or OH groups due to Li^+^. Maximum adsorption of different proteins was found at different lithium concentration, such as FN and BSA. Therefore, it is possible to selectively regulate proteins uptake on the surface and, consequently cellular response. Besides increased protein adsorption, ion presence in a biomaterial is beneficial due to eventual ions release. Co-implantation of Mg and Zn and their release as ions improve adhesion, proliferation, and motility of human gingival fibroblasts [161]. Bridging effect with proteins was confirmed also for trivalent ions such as Fe^3+^ [162], with benefits both in vitro and in vivo. Calcium ions showed further improved protein adsorption, also with respect to other divalent ions such as Mg^2+^ and Sr^2+^ [155,156,163]. Thanks to specific Ca-binding site on some proteins, such as laminin, osteopontin, which is a major non-collagenous bone protein [156], and BSA [163], they adsorb in higher amount on Ca-containing surfaces. Protein adsorption and, more important regarding implants, osteointegration were enhanced also by doping of titanium materials with phosphate ions on TNT [164] or on hydrothermal treated cp-Ti [158]. Growth of TNTs on Ti-Zr-Sn-Mo-Nb alloy resulted into sparse nanotubes due to alloying elements. Spacing between TNTs increased both protein adsorption and rat primary osteoblasts adhesion, proliferation, and activity [165].

All the doping treatments result also in morphological modification of the surface, which may also have a strong effect on protein adsorption. In fewer cases, mainly in the case of doping with monovalent ions such as Na^+^ [166], researchers found that morphology had a stronger effect than surface chemistry. Monovalent ions do not possess bridging capability towards proteins. Still, many more studies showed how ions presence in titanium-based biomaterials improved biological properties beyond surface nanostructurations [157,163,164].

### 3.4. Grain Size and Crystalline Phase 

Among the factors that influence surface properties such as wettability and surface energy, grain size, and crystalline form of the oxide layer on titanium surface play a major role. It is known that ultrafine-(UG) and nano-grain (NG) metallic surfaces show beneficial behavior with respect to coarse-grain structure. On 301LN stainless steel, grain size of a few nanometers was able to improve BSA adsorption and murine pre-osteoblast cells response [167]. Similar findings, along with improved mechanical properties were obtained on stainless steel 316L [168]. The effect of grain size on protein adsorption has been investigated by several studies, including several types of titanium-based materials such as cp-Ti [169,170], Ti64 [171], and titanium alloy [172,173]. Literature about the effect of nanocrystallization on protein adsorption is not in good agreement. Still, it is important to keep in mind that different results may arise from very different factors, such as surface chemistry, protein concentration in solution, and adsorption conditions. NGs on titanium were mainly obtained by surface mechanical attrition treatment (SMAT) or severe plastic deformation (SPD). The former treatment consists of bombarding the material surface with hardened steel balls [169,172]. The latter is obtained by mechanical stresses such as hydrostatic extrusion [170], sliding friction treatment [171], or high-pressure torsion [173]. All authors assessed that both treatments result in an increased volume of grain boundaries (GBs). They are highly defective sites that contributes to increase the surface energy and the hydrophilicity of titanium-based materials. Usually this resulted in augmented protein adsorption. Bahl et al. [169] applied SMAT to cp-Ti, obtaining nano-grains on the surface. Contrary to other studies, nanocrystallization obtained by SMAT decreases BSA adsorption due to changes in electronic and physicochemical properties of the oxide. Still, this is beneficial for attachment and proliferation of human MSCs (hMSCs) and also improved material hemocompatibility thanks to a reduced platelets attachment and corrosion resistance. Corrosion of metallic implants can be enhanced by proteins in solution [174]. Contrary to the adsorption behavior observed by Bahl, Kubacka et al. [170] observed an increase in BSA adsorption on cp-Ti after nanocrystallization through SPD. They found that adsorption from FBS results in increase of BSA uptake and in reduction of FN. GBs are regions where atoms are prone to be charged, resulting in increase of the acid-base component of the surface energy that is related to adsorption through electrostatic interactions. Thus, authors claimed that in this way non-specific protein adsorption, as BSA, is enhanced. Anyway, higher FN adsorption from FBS, along with VN was obtained on nano-grained Ti64 and Ti-Nb-Mo-Sn-Zr alloys. Huo et al. [171] ascribed enhanced protein adsorption on treated Ti64 to the smaller contact angle of NG surface with respect to coarse-grain and to higher surface energy. Thanks to a greater amount of RGD-containing proteins, these surfaces develop a suitable microenvironment for osteoblasts. β-alloy Ti-Nb-Mo-Sn-Zr subjected to SMAT treatment was found to adsorb twofold more FN and VN than the untreated surfaces [172]. Interestingly, the authors claimed that enhanced cell behavior on these surfaces is also related to proteins being adsorbed in a more active state with respect to coarse-grain surface. RGD groups are better exposed for cell attachment. Similar results were also obtained on Ti-Ni alloy subjected to high-pressure torsion [173]. VN adsorption increased more than BSA. Furthermore, Ni release was found to be hindered after SPD. 

**Figure 7:**
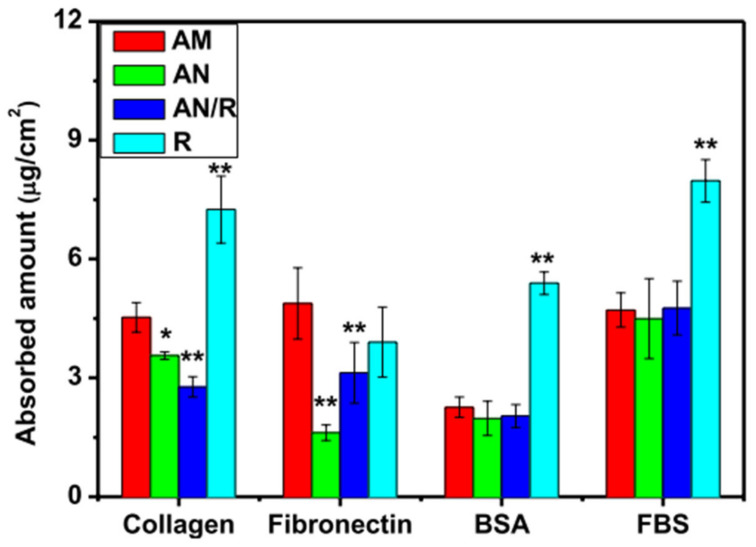
Adsorption of different proteins on Titania Nanotube (TNT) substrates with different crystalline phase: AM, amorphous; AN, pure anatase; AN/R, mainly anatase with rutile presence; R, pure rutile. Statistical difference by ANOVA: **ρ < 0.01 and *ρ < 0.05. Reprinted with permission from ref. [177]. Copyright 2019, King Abdulaziz City for Science and Technology.

Along with grain size, the crystalline phase of titanium surface oxide layer also plays a fundamental role in determining protein–surface interactions. Different titania phases, such as amorphous, rutile, and anatase, and their orientation change surface properties and protein adsorption. TiO_2_ phase is easily controlled through heat treatment: By increasing the treating temperature, amorphous titania is transformed into anatase at first and then to rutile, at about 600 °C [175]. The effect of different crystalline phases of titanium on protein adsorption has been investigated largely on TNT substrates [126,175,176,177]. Native oxide on flat cp-Ti was turned from amorphous to mainly anatase by annealing, showing almost no differences in the adsorption of BSA and FIB [126]. Nevertheless, the crystalline phase has different effects when adsorption from different proteins is investigated. Gong et al. [176] and Li et al. [177] agreed on the fact that anatase showed the lowest adsorption of COL I and FN compared with amorphous titania and rutile, as possible to see in Figure 7. The latter has the highest adsorption capability. On the other hand, adsorption of BSA or FBS were increased by higher annealing temperature [175]. 

Phase transformation highly affect the number of hydroxyl groups on the surface: Anatase has fewer OH groups than amorphous TiO_2_ and rutile has the highest number of all [175]. OH groups are fundamental to drive protein–surface interactions, in particular, basic OH groups can promote protein adsorption, and amorphous titania as more of them with respect to anatase [178]. Furthermore, anatase phase is more negatively charged than non-crystalline oxide or rutile, thus less proteins are adsorbed due to electrostatic repulsion [177]. Raffaini and Ganazzoli [179], through molecular modelling, observed that, among titanium oxide polymorphs, anatase provided the highest interaction energy for both BSA and FN. After initial contact, where the adsorption is driven by dipolar and dispersive interactions, both proteins tend to spread on the surface, in order to maximize amino acid residues interacting with the surface. BSA was found to do that on both anatase and rutile, while FN was more compact onto anatase. Higher crystallization obtained by heat treatment was beneficial for protein adsorption also on hydrothermally grown rutile nanoneedles [180]. Beside crystalline phase, also orientation of crystals may affect how proteins arrange on the surface. Molecular dynamic (MD) study allows to investigate protein adsorption by changing crystal’s Miller indexes. Myoglobin adsorbs on rutile (1 1 0) or (0 0 1) faces with different orientation [181]. Due to electrostatic repulsion, the HEME group is away from the oxygen rich (1 1 0) rutile face, while it is closer to the (0 0 1) one. Keller et al. [182] proved the effect of anatase orientation on conformation of adsorbed fibrinogen. Low SFE facets, such as the {1 0 0} family, behave as hydrophobic surfaces, favoring protein–protein interactions and formation of FIB networks. (1 0 1) and (1 1 0) crystals have higher hydrophilicity, the latter due to higher surface polarity, and favor adsorption of proteins in a globular-like shape. Globular conformation of FIB may reduce the inflammatory response to a foreign body since it is more similar to its native state.

### 3.5. Surface Activation

UV-light or plasm activations are very well reported to be a way of improve biological activity of biomaterials surfaces [183,184]. Increase of surface activity is achieved by a three-step mechanism: Removal of hydrocarbon contaminants; induced surface hydrophilicity; change of the surface charge from negative to positive. Medical devices would probably be stored for a very long time before usage, up to 5 years [185], therefore removal of atmospheric contaminants is a priority. This will be discussed deeply later in Section 4.1. 

**Figure 8:**
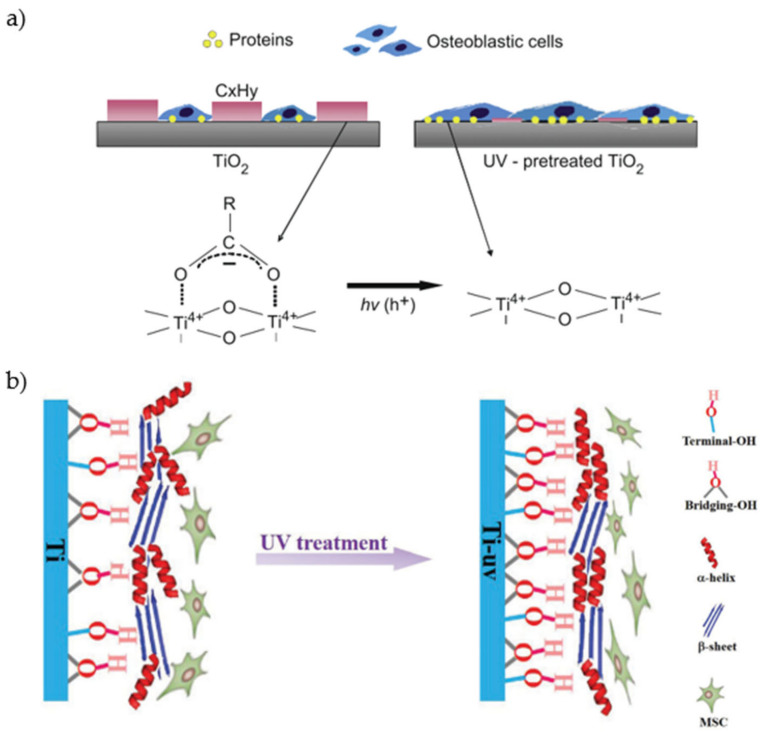
Schematic representation of UV effects on protein adsorption and cell attachment: (**a**) Removal of hydrocarbon contamination results in increased protein adsorption and osteoblast adhesion and spreading, adapted from ref. [186]; (**b**) effect of number and type of UV-generated OH groups on protein conformation and subsequent mesenchymal stem cell (MSC) proliferation, adapted with permission from ref. [187]. Copyright 2017 The Royals Society of Chemistry.

Protein adsorption on cp-Ti, in particular of BSA and FN was found to be strictly correlated to hydrocarbon level [186]. When contaminants are removed by UV, Ti^4+^ sites are exposed, increasing interaction with both protein and cell (Figure 8a).

Hydrophilicity and positive surface charge of UV-activated surface arise from the same physiochemical modifications of TiO_2_ layer, formation of oxygen vacancies, and terminal OH groups. Exposure to UV-light promotes an electron from the valence band to the conduction band. This causes a reduction of Ti^4+^ to Ti^3+^ and, as a consequence, oxygen vacancies are formed [183,188]. Other than increasing positive surface charge, Ti^3+^ are favorable sites for water dissociation, leading to generation of terminal OH groups [187]. Positive surface charge is also promoted by the basic behavior of UV-generated OH groups [189]. On titania nanoparticles, it was proven that basic hydroxyls can form hydrogen bonds with -NH_3_^+^ groups on proteins [178]. Electrostatic nature of protein adsorption enhancement was confirmed by Hori et al. [188]. They observed that more BSA adsorbed onto UV-activated surface from solution at pH 7 but a smaller increase was found at pH 3, compared with untreated Ti. At pH 7, both BSA and Ti are negatively charged. Thus, UV-generated positive charges can attract albumin molecules. At pH 3, BSA is below its IEP, as untreated Ti. Therefore, UV-activation is not as effective. Conformation of proteins is also affected by surface UV-activation, in particular by the terminal OH groups. Yu and coworkers [187] observed an increase of α-helix and a decrease of β-sheet contents in albumin, with respect to adsorption on an untreated surface. They discussed that these conformational changes can be related to increased osteogenic differentiation of MSCs(Figure 8b). Remarkably, while cell adhesion and proliferation on UV treated surfaces are increased, bacteria colonization of surface was hindered [190,191]. 

Different plasma system can be used in order to obtain activation of the surface: Different kinds of glow discharge plasma, such as atmospheric (APGD) [192], radio frequency (RFGD) [193], vacuum [194]; nonthermal atmospheric pressure plasma (NTAPP) [184]; or argon atmospheric pressure dielectric barrier discharge (APDBD) [195]. As well as UV treatments, plasma can increase protein adsorption thanks to the removal of hydrocarbon contamination [184] but, unlike UV, surface charge become more negative [184,193]. Specifically, employing NTAPP creates -COOH, -OH, and NH_2_ groups on the surface [39]. Oxygen-containing groups can generate reactive oxygen species during plasma treatments [184]. NTAPP treatments were found to have analogous effect to UV surface activation in terms of reduction of Ti surface negative charge and adsorption of BSA [196]. To the authors best knowledge, plasma effect was mostly investigated on fibronectin adsorption. Noticeably, FN adsorption was selectively increased in case of single protein solution [39,193] and when mixed to other proteins such as BSA [197] or even from plasma serum [194,198]. FN, as an adhesive protein, is beneficial for cell attachment and spreading *per se*. On plasma-treated Ti, negative plasma-induced charges affect FN conformation, promoting a more bioactive configuration of the protein on the surface. Integrin-binding sites on FN, namely the tripeptide sequence RGD, are more exposed due to conformational changes of the proteins. Thus, interactions with α_5_β_1_ integrin on cells are promoted, increasing osteoblast spreading and differentiation [39,193]. Controversy, Santos et al. [199] observed that low-pressure glow discharge plasma did not affect the total amount of adsorbed HSA, IgG, or LAM, nor the adsorption isotherms, when single protein solutions were used. Instead, plasma treatments affected the layer composition of proteins adsorbed from a mixture of the three of them. Adsorption of HSA and LAM were selectively increased and decreased, respectively, while IgG was not changed.

UV photoactivation and plasma treatments are effective ways to promote protein adsorption and surface properties in general. Since surface morphology is not modified, these techniques can provide useful information on adsorption mechanisms, by isolating the effect of surface charges and functional groups. Despite not being addressed as the main adsorption driving force, electrostatic interactions have been proved to play an important role, in particular regarding selective adsorption of proteins and their biological activity.

## 4. External Parameters Affecting Protein Adsorption on Titanium Surfaces

### 4.1. Aging and Storage: Contamination of Titanium Surfaces

Biological properties of titanium need to be preserved even through the long-lasting storage of the biomedical devices, up to five years. Implants and dental screw are usually enclosed in a sterile gas-permeable packaging, which keeps the contents sterile but allows contamination of the surfaces by the carbonaceous organic impurities in the atmosphere [185,200]. Very recent molecular dynamic studies performed by Wu et al. [201] demonstrated that carbon contaminants expose C-H bonds, thus greatly reducing surface polarity and dipole–dipole interactions with proteins. Protein adsorption drastically decreases after four-week storage when titanium is placed in sealed container [202]. Proportional correlation between increased WCA and reduced protein adsorption was observed. The same authors observed that inclusion of divalent ions like Ca^2+^ within Ti surface through chemical treatments may hinder depression of bio-properties due to aging [203]. After four weeks, BSA adsorption, and rat BMCs attachment resulted in being higher with respect to untreated surface. Therefore, it is necessary to limit surface contamination of implants during their shelf-life. As described in the previous paragraph, plasma treatments are an effective way to remove carbon contaminants form titanium surface. Despite being very efficient in removal of carbon contaminants, UV and plasma might be time-consuming processes and require delicate equipment. Miki et al. [204] found that simple cleaning of titanium devices with electrolytic reducing ionic water, which has high OH^−^ concentration, led to similar results in protein adsorption compared with UV. Bone contact is also much higher than the control surfaces. This process can be easily performed in a generic dentistry facility, for example. In the past 10 years, great efforts have been made by researchers to find a suitable storage method. It shall maintain intact the biological properties of a newly manufactured Ti surface and it needs to withstand sterilization. Storage in wet conditions seems to be the most promising way [205,206]. Choi and co-workers observed that soaking in distilled water may retain properties of titanium surface after UV and plasma activation for periods up to eight weeks [206]. UV treatment and wet storage on SLA-modified Ti surface allow to obtain the best results, in terms of protein adsorption and murine osteoblast cells adhesion. Interestingly, storage in water is not only suitable for avoiding carbon contamination, but also it is capable of maintaining the more positive surface charge of the UV-treated titanium [200]. Protein adsorption and cell adhesion can be even increased upon storage by using ion-containing solution [205]. Ca-containing solution can benefit from the protein bridging effects of adsorbed ions and the cellular affinity for these ions. Vacuum storage proved to retain biological activity of alkali-heat treated titanium eve after one year [185]. Wilhelmi et al. [50] confirmed, through time of flight secondary ions mass spectroscopy (Tof-SIMS) analysis, that the maximum adsorption of BSA on cp-Ti is obtained for solution at pH 5.2, and decrease with increasing pH values. This confirm that protein–protein latera interactions play a major role in adsorption mechanisms. 

### 4.2. Influence of the Solution: pH, Temperature and Ions

**Figure 9:**
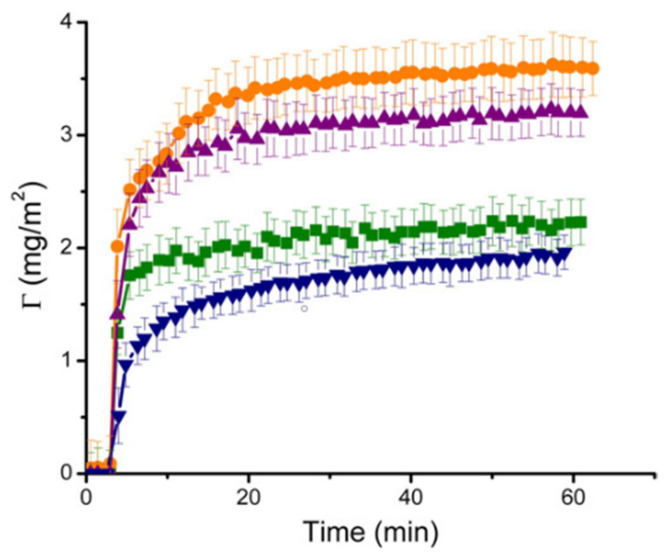
BSA adsorption on TiO_2_ thin film at different pH values: 3.55 (■), 4.60 (●), 5.60 (▲), and 7.51 (▼). Reprinted with permission from ref. [208]. Copyright 2009 Elsevier B.V.

As discussed in paragraph 2.4, the parameters of the protein solution have a major role in determining various aspect of protein adsorption, such as amount of the protein adsorbed, surface–protein interactions, and adsorption kinetic. The pH value determines charge distribution on both the surface and protein [20]. As a consequence, protein–surface interactions may vary to a great extent by changing pH. This fact also reflects to loosely bound proteins. At pH close to the IEP of a protein, repulsive interactions between BSA molecules are at the lowest, therefore the loosely bound portion of proteins adsorbed is increased [207]. The highest adsorption was observed near the IEP of the BSA, as it is possible to see in Figure 9 [208]. The mechanisms that can explain the pH effect on protein adsorption on Ti was proposed by Imamura et al. [209]. At pH around 4, that is below the IEP of Ti, acidic residues in the proteins, with COO^-^ groups, are attracted by OH^+^ groups on surface. Above pH = 5, the functional groups on the Ti surface turn negatively charged and can interact with -NH_2_^+^/-NH=NH_2_^+^ groups of the amino acids. They also observed that thickness of the adsorbed protein layer may vary by changing pH. Similar behavior was observed also for titania. 

Still, due to the high adsorption even under adverse electrostatic condition, namely at pH 3.55 and 7.51, the authors claimed that the main driving force for albumin adsorption on titania is hydrophobic interaction. LYS adsorption was also observed to be strongly dependent on pH [210]: When titania is positively charged, at pH lower than 5, almost no adsorption was observed due to electrostatic repulsion, as expected, because at this pH value, LYS is positively charged. In this study, correlation between protein uptake and temperature was also discussed. Increased temperatures lead to higher amount of adsorbed proteins. Combined effect of pH and temperature was studied by Kopac et al. [44]. By fitting adsorption isotherms with Langmuir or Freundlich curves, they observed that the highest adsorption of BSA onto titania can be obtained at 40 °C and pH 4. These data show that adsorption from different solution may results in very different protein layer on the surface of biomaterials. 

Ions dissolved within the protein solution compete with protein for interacting with the surface and hinder or elicit protein adsorption. Phosphate ions can easily adsorb on titanium surfaces [56] and they alter BSA adsorption kinetic and conformation on TiO_2_ [211]. Positive mono- and divalent ions are electrostatically attracted by the negative charges on titanium, subsequently mediating the interaction between the surface and proteins. Monovalent ions, such as K^+^ and Na^+^, do not influence to a great extend protein adsorption [59] since once their single positive charge is attracted by the surface, they have no more for proteins to be attracted. On the other hand, divalent ions, Ca^2+^ and Mg^2+^ in particular have a bridging effect toward proteins thanks to spare positive charges after interaction with titanium [212,213]. Kohavi et al. [59] observed that electrostatic interactions may play a major role than surface wettability on protein adsorption. Adsorption of HSA and FN was enhanced by prewetting Ti64 surfaces, with solutions containing divalent ions or not. Wetted surface were hydrophilic, and non-wetted ones were hydrophobic. After being dried, surfaces turned hydrophobic again, and adsorption was still enhanced on the samples that were wetted with Ca^2+^ containing solutions. The interplay between pH and ions dissolved in determining the electrostatic interactions between proteins and surfaces was well described by Hori et al. [188]. Around physiological pH, when both titanium and albumin are negatively charged, divalent ions are effective in increasing protein adsorption. At pH 3, below the IEP of surface and protein both, ions did not alter amount of adsorbed BSA. In the same study, it was also found that anions, as Cl^-^, can mask UV-generated positive charges and annihilate the beneficial effect of UV treatments. The fact that ions co-adsorption can reduce benefits of positive surface potential on the adsorption of proteins was recently confirmed [214].

After all these considerations, it is possible to state that attention must be paid to the solution parameters, in particular when discussing protein adsorption and comparing results from different studies.

### 4.3. Protein Concentration in Solution

Human plasma and biological fluids in general contain proteins in a very high concentration. The amount of proteins in human plasma is in the range of 60–80 mg/mL [215]. It is not trivial to reproduce this high concentration in laboratory experiment, mainly due to the high cost of proteins and their availability for purchase. Thus, researchers investigating protein adsorption used to lower protein concentration in solution, from some mg/mL [216] down to small fraction of the biological one [145], for example BSA was employed in concentration ranging from 0.4 to 4 mg/mL while its biological concentration is reported to be as high as 33–52 mg/mL [217]. As pointed out by Hemmersam and co-workers [218], adsorption of proteins from low concentrated solution has a stronger dependence from the substrate than what happens using higher protein concentrations. Using quartz crystal microbalance (QCM) analysis, they found that adsorption of FIB from 0.03 mg/mL solution to Au-, Ti-, or Ta-sensors showed larger differences, both in layer structure and protein amount, than in the case where 1 mg/mL solution was used. In the former case, FIB molecules had time to spread on the surface and to interact with it using both αC and D domains, adhering more strongly. In the latter case, adsorption rate is too fast for this to happen. Strong denaturation of proteins adsorbed from low concentrated solution was observed also for FN on Ti64 [219]. Protein unfolding is also hindered by surface hydrophilicity obtained through UV activation. Reducing the amount of proteins used during an experiment, paying the price of be further away from real physiological conditions, may be necessary to appreciate the influence of the material features on adsorption mechanisms. Beneficial effect on protein adsorption obtained by Argon plasma or UV treatments of pure titanium turns insignificant when adsorption was carried out using 10% FBS solution instead of 2% one [220]. Researchers need to bear in mind that surface effects observed using solution with a very low content of proteins can be reduced, different, or, in the worst case, annihilated in case of the real, biological fluids. This fact applies also for properties and characteristics of the protein adsorbed layers.

## 5. Protein Co-Adsorption and Competition for the Surface

Human plasma contains about 3020 distinct proteins [221]. As for protein concentration, it is nearly impossible to replicate this enormous complexity on a lab scale. In addition, it would be extremely complex to understand adsorption mechanisms and the specific role of the biomaterial surface in it. Thus, most of researchers conduct their experiments using a single protein solution, as shown previously. Unfortunately, it is not possible to predict the adsorption of proteins from complex mixture just knowing how it happens from the single protein solutions. Researchers tried to expand knowledge of the protein adsorption on titanium-based biomaterials by mostly using binary protein mixture or subsequent adsorption.

**Figure 10:**
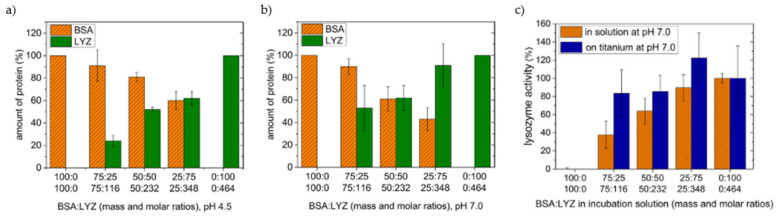
Adsorption on cp-Ti from BSA-LYS mixture: Relative amounts of adsorbed proteins form mixtures with different ratios (BSA: LYS 100:0, 75:25, 50:50, 25:75, 0:100) at different pH, 4.5 (**a**), 7.0 (**b**) (amount is expressed as percentage of adsorbed protein from a pure solution); LYS enzymatic activity, relative to pure LYS solution, in mixture with BSA and after adsorption from same mixture (**c**). Adapted with permission from ref. [222]. Copyright 2016 Elsevier B.V.

Adsorption of BSA on cp-Ti was found to be enhanced when obtained from BSA-LYS containing solutions [222,223]. BSA^—^LYS^+^ agglomerates can form in solution and adsorb on the surface, thus increasing the total mass of adsorbed proteins (Figure 10). Relative amounts of adsorbed proteins are influenced both by solution composition and pH, as possible to see in Figure 10a,b. Interestingly, residual enzymatic activity of LYS is not much influenced on protein content in solution [222]. BSA, due to its larger mass, cannot be displaced by LYS in case of sequential adsorption, thus limiting LYS amount on the surface [223].

Physiochemical characteristics of surfaces have a strong role in determining protein competition for the surface. Hydrophilic titanium surfaces, such as SLA-treated substrates, have been found to promote FN adsorption during competition with albumin, even when a biological BSA:FN ratio of about 100:1 is maintained in the solution [224]. The first protein to adsorb on the surface can inhibit sequential adsorption of other proteins [225], but higher affinity with the surface can result in protein displacement and substitution [225,226]. Felgueiras et al. [226] showed that FN and COL I can block sequential albumin adsorption on Ti64 due to their larger mass, and that they are able to displace albumin when adsorbed as second proteins. Interaction between FN and COL I are dependent on the kind of the surface. As in the case of LYS, albumin can form complexes with COL I in solution resulting in higher number of proteins adsorbed with respect to the single protein solutions. Contrary to these results, BSA was found to be able to displace larger proteins such as FIB or FN on TiO_2_ surfaces [227] due to higher affinity for the surface. On a pre-existing BSA layer, FIB and FN forms a layer on the albumin instead of displacing it. 

Being quite simple, binary protein solutions are still not very much representative of actual biological fluids. Some researchers moved further on in complexity of systems by investigating through proteomic analysis the exact composition of protein layers adsorbed on several titanium surfaces from real and whole biological fluids such as plasma [221,228,229] or saliva [230,231,232]. Among the thousands of proteins present in human plasma, the most adsorbed was FN, followed by albumin, alipoprotein, and fibrinogen [221]. From saliva, which contains about 750 different proteins, less than half of them were found on titanium [232], mainly amylase and lysozyme [230]. The effect of surface modification on the protein pellicle composition was also evaluated. In case of adsorption from saliva, very low specificity was observed for different titanium surfaces, smooth, SLA-treated, and SLA-treated+stored in ionic solution [231]. On the contrary, differences were observed between smooth and SLA surfaces by using human serum [229]. One hundred and eighty-one and 162 proteins were identified on smooth and blasted/acid-etched surfaces, respectively. Proteins adsorbed onto smooth Ti are involved in a higher number of biological pathways, such as clotting, cytokines-mediated inflammation response, integrin signaling, and glycolysis, the latter being absent on SLA-treated titanium. SLA treatments were also found to affect the proteome on Ti-Zr alloy, from both plasma and saliva [233]. Adsorption from saliva resulted in 389 common adsorbed proteins, 40 adsorbed uniquely on the machined samples, and 14 on the SLA treated ones. The proteome from blood plasma was much more similar with only three unique proteins, on both machined and SLA surfaces, and 145 common proteins. Even though UV activation of the surface has been reported to improve adsorption of proteins [188], proteomic analyses found that light treatment on cp-Ti, hydrothermally coated with nano-structured TiO_2_, depress proteins adsorption from plasma [228]. Much lower content of FIB, immunoglobulins, and other proteins were found on pre-activated surfaces. Authors addressed this decrease to a mutual combination of surface properties: Roughness, charge, intrinsic, and photo-induced wettability. Reduction of inflammation-promoting proteins such as immunoglobulins and FIB may be beneficial for osseointegration. Even if adsorption from complex biological fluids can be more significant than adsorption from a single protein solution for understanding the fate of biomaterials, this might not be still enough. Jager et al. [234] studied the composition of the protein layer formed onto explanted hip implants. They found that proteome formed onto a titanium implants is different with respect of the one that forms from plasma. Among the 2802 unique proteins founded on the implant, cell-free hemoglobin was the most abundant, almost two-fold albumin. Most of them were of intracellular origin and, interestingly, fibronectin was absent. 

Adsorption from single protein solution can be useful for a preliminary understanding of how the different surface features may interact with biological fluids after being implanted. Anyhow, it is evident that this is not sufficient and it is quite necessary to test protein-biomaterials interactions using complex solutions. 

## 6. Methods for Investigating Protein Adsorption on Titanium-Based Materials

During the past years, researchers have developed and optimized a huge number of experimental techniques in order to overcome the challenges of investigating adsorption of proteins on surfaces with very different features. Characterization techniques for proteins adsorption, and biomolecules adsorption in general, are extensively reviewed elsewhere [235,236]. Here, a brief overview is reported of the techniques used in literature specifically for characterizing adsorption on titanium-based biomaterials. Different aspects of proteins adsorption need to be targeted by characterization techniques, for instance protein quantification, conformation of the adsorbed proteins, protein–surface interactions, and protein type recognition. Experimental techniques will be described according to the information that they can provide about the protein adsorption phenomenon, their advantages, and drawbacks in the characterization of biomaterials, as summarized in Table 2.

One of the first issue when studying protein adsorption is to quantify the molecules adsorbed onto the surface. Two main strategies are at the disposal of researchers to perform a direct quantification of the adsorbed protein: Unlabeled proteins and labeled proteins. In the latter case, proteins can be labeled with iodine isotope ^125^I [144,145] or with fluorophores, as rhodamines [74,100]. The use of fluorescent markers allows also to image the protein layer. Quantification of proteins can be achieved with label-free techniques. Bicinchoninic acid assay (BCA) is one of the most employed analytical assay for the quantification of proteins [98,101], alongside the Bradford method [224]. With these techniques, adsorbed protein can be determined by removing the proteins form the material surface or by measuring the remaining concentration in the uptake solution. Underestimation of the adsorbed proteins may occur if proteins are not completely detached from the surface. Protein concentration in solution can be evaluated thanks to the Lambert-Beer law, using a wavelength of 280 nm [81,117] Other techniques may be adapted from biochemistry in order to quantify and recognize adsorbed proteins. In some studies, the use of gold-labeled [131] or fluorescent-marked antibodies [107] is reported, which allows to target specific proteins and quantify or even image them. With these methods, it is also possible to search specific proteins within mixture. ELISA is another suitable and widespread method to evaluate the amount of very specific proteins adsorbed ono the surface [104,128,129,152]. Determination of the specific composition of the protein layer formed onto a surface can be performed using different methods: Gel electrophoresis is commonly used in various forms, such as Western blot or sodium dodecyl sulphate–polyacrylamide gel electrophoresis (SDS-PAGE) [130,162,199,230], to separate and recognize different proteins; liquid chromatography electrospray ionization tandem mass spectrometry (LC-ESI-MS/MS) is another largely used method to identify proteins within a complex layer [90,221,231]. Conventional techniques for surface chemical analysis in material science are also valuable for evaluating presence of protein on a surface. XPS [75,123] and Tof-SIMS [50,128,223] can detect elements or functional groups characteristic of proteins and also identify specific proteins. Furthermore, XPS can also be employed for investigating protein–surface interactions [114,148]. X-ray wavelength dispersion spectroscopy (WSD) was also reported as effective for detect adsorbed proteins in a large concentration range, from ng/cm^2^ to µg/cm^2^ [76,147]. Imaging and qualitative detection of proteins onto a surface can be also performed by atomic forces microscopy (AFM) [58,110,143]. It is possible to image single proteins or agglomerates on the surface [58] or, thanks to appropriate tip modifications, it is also possible to measure the interaction forces between the proteins and surfaces [82]. Distribution of proteins onto a surface can be imaged thanks to the use of confocal laser scanning microscopy (CLSM) coupled with use of fluorescent-labeled proteins [95,100]. Qualitative evaluation of protein adsorption can be performed directly visualizing the adsorbed layer by transmission electron microscopy (TEM) [180]. Zeta potential measurements is usually applied for nanoparticles or particles in suspension [196], but it is also possible to obtain titration curves on bulky samples [54]. IEP shift and curve shapes can provide information on the surface coverage and protein conformation. Real-time monitoring of adsorption process can be obtained by QCM. With this technique, proteins adsorbed onto the surface can be weighted [112]. Different QCM set ups allow to collet further information: QCM with dissipation (QCM-D) allows to measure the dissipation of energy in the adsorbed layer, thus providing information about the flexibility and the water content of the adsorbed layer [67,126,227]; QCM can be coupled with electrochemical and impedance measurements (EQCI) [66]. Mechanisms of protein adsorption are very complex to be characterized. The family of spectroscopic techniques comprehend several powerful methods, which allows to investigate protein secondary structure, layer thickness, and chemical environment of certain amino acidic residues. Protein conformation, in terms of α-helix, β-sheet, and random coils content, can be determined by Fourier transform infrared (FTIR) spectroscopy, in particular in Attenuated Total Reflection (ATR), thanks to deconvolution of Amide I band [113,216]. Amide I and II band intensity can also provide quantitative information about adsorbed proteins [88,123]. Raman spectroscopy is a technique mostly applied for studying adsorption on nanoparticles [36,237], which was also reported to be used, with 2D-correlation analysis, for adsorption on bulk materials [71]. The evolution of the adsorbing layer can be monitored in situ by spectroscopic ellipsometry by measuring the thickness of the layer, according to the variation of the ellipsometric angles Δ and Ψ [86,208,209]. Some amino acids are intrinsically fluorescent, tryptophan and tyrosine in particular, and their emission is sensitive to the chemical environment around them. By choosing a suitable Δλ, synchronous fluorescent spectroscopy (SFS) can be applied to monitor the conformation of proteins around such residues [113,114,115]. The electrochemical behavior at the protein–surface interface can provide information about the evolution of the layer and protein–surface interactions. This information can be obtained by electrochemical impedance spectroscopy (EIS) performed in protein-containing solutions [57,61,169,212]. Information about the denaturation of proteins after adsorption can be obtained by exploiting circular dichroism (CD) analysis [187].

**Table 2:** Characterization techniques commonly used for protein investigation on titanium-based surface. The output about protein adsorption, the kind of substrates that can be analyzed, the possibility of in situ (without protein detachment) and real-time measurement, and main advantages and drawbacks are reported.

Technique		Output	Substrate	In Situ/Real Time	Advantages	Drawbacks	References
Labeled proteins	^125^I-labeling	Quantification	Any	Yes/no	Direct quantification	Change of protein properties, handling issues	[144,145]
	Fluorescent labeling	Quantification and imaging	Any	Yes/no	Direct quantification, competitive adsorption evaluation	Change of protein properties, expensive reagents	[74,80,100,116]
UV-vis spectroscopy	BCA	Quantification	Any	No/no	Low cost, large range of concentrations	Protein detachment needed	[98,101,114,115]
	Bradford assay	Quantification	Any	No/no	Low time consume	Protein detachment needed, sensible to surfactant	[88,155,224]
	Spectrophotometry (λ = 280 nm)	Quantification	Any	No/no	No reactant needed	Protein detachment needed, inaccurate with complex samples	[81,117]
Labeled antibodies		Quantification, protein recognition and imaging	Any	Yes/no	Targeting of specific proteins	Time consuming, specific reagents	[94,107,131,173]
ELISA		Quantification and protein recognition	Any	Yes/no	High specificity	Time consuming, specific reagents	[104,128,129,152]
Gel electrophoresis	Western blot	Quantification and protein recognition	Any	No/no	No toxic chemicals	Sample preparation, poor band separation	[102,130]
	SDS-PAGE	Quantification and protein recognition	Any	No/no	High sensitivity, small samples needed	Poor band resolution, toxic chemicals	[109,230]
LC-EIS-MS/MS		Proteomic analysis	Any	No/no	High specificity and sensitivity	High costs	[229,233,234]
XPS		Quantification, protein-surface interaction	Any	Yes/no	High sensitivity, simultaneous evaluation of surface chemistry, depth profiling	No absolute quantification, complex data analysis	[110,114,133,212]
Tof-SIMS		Quantification, protein recognition	Any	Yes/no	High sensitivity, possible orientation and conformation analysis, depth profiling	No absolute quantification, complex data analysis	[50,128,223]
WSD		Quantification	Any	Yes/no	Sensitive to a wide range of protein surface concentration	Thorough calibration needed	[76,147]
AFM		Imaging, adhesion forces, conformation	Flat substrates	Yes/no	High resolution, customizable tip	Low throughput, time consuming	[58,82,110,143]
CLSM		Imaging, relative quantification	Any	Yes/no	High resolution, 3D distribution into surface features	Expensive reagents	[95,100]
TEM		Imaging, thickness measurement	Any	Yes/no	Direct visualization of protein layer	Complex sample preparation	[180]
Zeta potential		Adsorption evaluation, protein conformation	Powder or planar samples	Yes/no	Simple measurement	No protein recognition, preliminary information needed	[54,78,228]
QCM		Quantification, viscoelastic properties of layer, changes in conformation	Sputtered sensors	Yes/Yes	High sensitivity, real time measurement, possibility to change the uptake solution	Co-adsorbed solvent weighted. Mass calculation affected by energy dissipation	[67,112,226,227,238]
FTIR (ATR)		Secondary structure, relative quantification	Planar samples	Yes/no	Very specific protein band	Not highly sensitive, data deconvolution needed	[113,114,216]
Raman spectroscopy		Secondary structure, relative quantification	Any	Yes/no	Very specific protein band	Not highly sensitive, complex data interpretation	[71]
SE		Layer thickness measurement	Flat surfaces	Yes/yes	High sensitivity, low cost, fast measurement	Difficult optical modeling of rough and structured surfaces	[86,208,209]
SFS		Protein conformation	Any	Yes/no	Sensitive, high selectivity towards specific amino acids	Possible instrument artifacts	[113,114,115]
EIS		Layer evolution, protein-surface interactions	Planar samples	Yes/yes	High sensitivity, possible to study adsorption in different condition	Complex modelling and data interpretation	[57,61,169,212]
CD		Protein conformation	Planar samples	Yes/no	Specific bands for secondary structures	Band deconvolution needed	[187]

## 7. Key Concepts

Protein adsorption is a fundamental step in the interaction of implantable biomaterials, such as titanium and titanium alloys, with the biological environment. The positive or negative outcome of tissue integration of an implant depends on the interplay between the body and the implant surface. How cells and bacteria adhere, proliferate, and compete is strongly dictated by the protein layer that forms on the device surface within the first minutes after implantation. The understanding of these phenomena is necessary to develop always better implants and to reduce possible adverse reactions. Thus, in past years, great efforts have been put to gain knowledge about the aspects that regulate proteins adsorption on titanium. A large variety of different surface–protein combinations have been investigated, including different type of titanium, titanium oxide, and titanium alloys, several kinds of surface treatments aimed to improve Ti osseointegration and a wide range of proteins in a simpler or more complex environment. Due to the enormous variability and complexity of the protein adsorption processes, a unique and fully agreed explanation of adsorption on titanium was not found in literature, some aspects being clearer than others. Impact of surface properties, such as roughness, morphology, chemistry, surface energy, wettability, and charge, need further investigation. The main effects of titanium surface features are summarized in Table 3.

Increased surface roughness in the micro scale seems to be capable of increasing the adsorption due to a greater number of active sites and features such as pores, nanotubes, or pits can accommodate proteins. On the other hand, no clear effect was found for nano-roughness. In this case, topography effect is mediated by other properties, such as charge or wettability. Electrostatic attraction may increase protein adsorption, while repulsion seems not enough to completely avoid protein binding with the surface. The role of wettability in adsorption is the most controverse. As a rule of thumb, proteins prefer to adsorb on hydrophobic surfaces, since water is more easily displaced from the surface and hydrophobic interactions between ammino acid residue and surface can be strong. In fact, this has been reported in some cases for adsorption on titanium surfaces. On the other hand, hydrophilic surfaces usually present more OH groups, higher surface charge, and SFE. These factors can promote surface–protein interactions, making adsorption favorable also on wettable surfaces. Furthermore, wettability can enhance solution-surface contact by turning it from a Cassie-Baxter to a Wenzel regime. These factors are able to promote protein adsorption against the generally accepted rule of thumb. The ongoing research on development of new and more bioactive surfaces had introduced more factors that can influence protein adsorption: The presence of ions within the oxide layer or of metals as alloying elements, the control over grain size, and surface activation treatments. All these features strongly change surface properties, namely wettability, hydroxylation, charge, SFE, roughness, making it less trivial to discriminate what features influence proteins adsorption and how. Conformation and orientation of adsorbed proteins are also heavily affected by surface properties in a non-unique way. Aspect ratio of surface features can change how proteins accommodate on the surface, higher hydroxylation may promote denaturation and spreading of certain proteins, while in other cases, OH groups increase wettability consequently reducing protein–surface interactions.

Besides, the poor standardization and use of testing protocols among researchers led to different conclusions about protein adsorption. The wide variety of protein concentrations, solution composition, and experimental methods make it very difficult to compare different works and to state if a system is an effective representation of the real adsorption process as occurring in vivo, within the human body. The complexity level of the system used can completely change how proteins interact with a surface, and scaling up from a simple single protein solution seems not to be an effective way to understand how materials behave when put in contact with biological fluids.

Today, knowledge about protein adsorption on actual implant surface is also limited by the fact that it is not trivial to find characterization techniques that can provide information about adsorption mechanisms on real surfaces. In the literature, a lot of techniques have been used to investigate protein adsorption on titanium materials (see Table 2), but some of them may not be appliable on a bulky and surface treated titanium sample because they need specific characteristics, such as surface flatness, planar specimen or surfaces need to be grown on the instrument sensors. These kinds of model surfaces may not be representative of the surface of a real implant. 

## 8. Conclusions

In conclusion, in the past 15 years, great efforts have been put into building deeper knowledge of how proteins and titanium-based biomaterials interact. Many different aspects of the complex adsorption phenomenon have been investigated by using a wide range of different surfaces, tailoring specific characteristics and exploiting adsorption environments ranging from a simple single protein solution to actual human biological fluids or even in human body. However, no generally accepted adsorption mechanisms have been found, with researchers sometimes being in disagreement about how surface properties or surface treatments affect protein adsorption. This can be ascribed to the absence of standardized and commonly used experimental protocols: protein concentration, adsorption condition and eventual rising methodology, all may alter the formation and evolution of the protein layer on materials surfaces. It is desirable that, in future, protein adsorption will be investigated through more common and shared methodology, obtaining comparable results. Furthermore, the researchers shall both study simple model systems and mimic, as close as possible, the physiological condition, as in case of protein concentration. This may be not trivial, but it allows comprehension of both very detailed aspects of the adsorption mechanisms and how actual implant surfaces will behave when employed as biomaterials. In parallel, further efforts have to be spent into development or optimization of the characterization techniques that can be applied on a wide range of materials in terms of sample shape and surface characteristics, mainly surface roughness. This is necessary for a correct evaluation of adsorption properties of biomaterials that may be actually employed as implants. Being extremely sensitive to surface features, protein–surface interactions may not be reproduced in an effective way on model surfaces. 

In any case, according to the literature reviewed here, surface modifications that enhance adsorption of proteins, in particular adhesive ones such as fibronectin and vitronectin, are generally the same, which are able to increase cell proliferation and to promote osteoblast differentiation and in vivo bone integration. Deeper and better comprehension of protein adsorption will allow also a more efficient design of biomaterial surfaces, chasing the perfect implant.

Twenty years have passed since Imamura and co-workers described protein adsorption on solid surfaces as “a common but very complicated phenomenon” [239], and in this time, thousands of papers addressing this issue have been published. Nevertheless, protein adsorption is such a complex matter that a fully and comprehensive explanation of it is still missing. Research groups need to put further efforts to enlighten hidden aspects of protein adsorption and to put an end to the “quest for a universal mechanism” [240].

## Figures and Tables

**Table 1:** Main parameters affecting protein adsorption on surfaces.

	Parameters	General Rules of Thumb
Surface	Topography/roughness	Higher surface roughness ≥ higher amount of adsorbed proteins
	Hydrophobicity (non-polar surfaces) Hydrophilicity (polar surfaces, with a net surface charge)-	Higher hydrophobicity ≥ higher amount of adsorbed proteins and denaturation degree; hydrophobic interaction as adsorption mechanism Different mechanisms of adsorption on hydrophilic surfaces: electrostatic, van der Waals, dipole-dipole; adsorbed water must be removed for adsorption
	Chemistry (functional groups, metal ions)	Influence on the surface charge
Protein	Amino acid chain	Affects structural stability
	Hydrophilicity/hydrophobicity	Surface charges and non-polar residues are always present; they can be differently arranged according to the environment; hydrophobic residues interact with hydrophobic surfaces
	Charge	Higher amount of adsorbed proteins at IEP
	Molecular weight	Small proteins adsorb quicker Large proteins replace the smaller ones and make stronger bonds with the hydrophobic surfaces
	Structural stability	Soft proteins change easier configuration and adsorb larger on hydrophilic surfaces; denaturation can enhance or reduce biological activity
Solution	pH	Affects surface charge of both proteins and surfaces
	Ionic strength	Adsorbed ions reduce repulsive effects among proteins; some ions compete with proteins for adsorption
	Protein concentration	Higher protein concentration higher amount of adsorption
	Protein mixture(single, binary or more complex)	Vroman effect
	Temperature	Higher temperature ≥ faster kinetics of adsorption

**Table 3:** Effect of titanium surface properties on protein adsorption (amount of adsorbed proteins, protein conformation on surface, and mechanism of protein–surface interaction) and impact of each feature on adsorption. ≈: no clear impact; ↑: mild impact; ↑↑: high impact; n.r.: effect not reported.

Surface Characteristic	Impact on Protein Adsorption	Conformation	Mechanism	Examples
Microroughness	↑	n.r.	Higher interaction area, physical adsorption	SLA surfaces adsorb fourfold more of albumin, fibronectin, fibrinogen and immunoglobulin vs. untreated surface because of roughness. Laser patterning increases adsorption of FIB.
Nanoroughness	≈	↑	Dependent on other characteristics. Aspect ratio of nanofeatures can influence protein conformation.	BSA aggregates into nanopores larger than its hydrodynamic radius with a strong interaction with the surface, while FN is too large. BSA/FIB adsorb as multilayer with stronger protein-protein interaction on nano-rough surfaces
Hydroxylation	↑↑	↑↑	According to the specific adsorbed proteins, OH can promote or hinder interaction with the surface	BSA adorbs through hydrogen bonding and proton transfer with interaction with OH surface groups. FIB adsorbs through positive charged αC domains. Rutile adsorbs more COL, FN and BSA than anatase or amorphous titania due to higher OH density
SFE	↑↑	n.r.	High surface energy, in particular the polar component, increases adsorption	Ti adsorbs larger amount of plasma proteins vs. other metals with lower SFE, but TiO_2_ adsorbs less proteins and in a weaker manner than other oxides with higher SFE. Ti adsorbs less basal lamina and salivary proteins than polymers for dentistry. Sandblasting with SiC induces higher SFE and preferential adsorption of FN. Laser patterning induces higher adsorption of FN by increasing the polar component of SFE. Nanograined surfaces have higher volumes of grain boundaries, which increase the SFE and adsorption of FN and VN
Charge	↑↑	↑	Can promote or limit protein adsorption, depending on charge of both surface and proteins	BSA is adsorbed in a lower amount on negatively charged surfaces while it is the opposite for histone that is positively charged. UV-generated positive surface can adsorb more BSA at pH 7, when the protein is negatively charged.
Chemistry (alloying metals, ions)	↑	n.r.	Increase protein adsorption, divalent ions in particular	TiNi alloys results in lower BSA (dependent on Ni content), FIB, and FN adsorption vs. cp-Ti. Ion-doped Ti has increased surface charge and protein adsorption because of bridging effect of divalent ions or specific chemical bonds (Ag)

## Data Availability

Not applicable.
